# Bacteriophage Therapy for Difficult-to-Treat Infections: The Implementation of a Multidisciplinary Phage Task Force (*The PHAGEFORCE Study Protocol*)

**DOI:** 10.3390/v13081543

**Published:** 2021-08-05

**Authors:** Jolien Onsea, Saartje Uyttebroek, Baixing Chen, Jeroen Wagemans, Cédric Lood, Laura Van Gerven, Isabel Spriet, David Devolder, Yves Debaveye, Melissa Depypere, Lieven Dupont, Paul De Munter, Willy E. Peetermans, Vera van Noort, Maia Merabishvili, Jean-Paul Pirnay, Rob Lavigne, Willem-Jan Metsemakers

**Affiliations:** 1 Department of Trauma Surgery, University Hospitals Leuven, 3000 Leuven, Belgium; baixing.chen@student.kuleuven.be (B.C.); willem-jan.metsemakers@uzleuven.be (W.-J.M.); 2 Department of Development and Regeneration, KU Leuven, 3000 Leuven, Belgium; 3 Department of Otorhinolaryngology, University Hospitals Leuven, 3000 Leuven, Belgium; saartje.uyttebroek@uzleuven.be (S.U.); laura.vangerven@uzleuven.be (L.V.G.); 4 Department of Neurosciences, Experimental Otorhinolaryngology, Rhinology Research, KU Leuven, 3000 Leuven, Belgium; 5 Department of Biosystems, Laboratory of Gene Technology, KU Leuven, 3000 Leuven, Belgium; jeroen.wagemans@kuleuven.be (J.W.); cedric.lood@kuleuven.be (C.L.); rob.lavigne@kuleuven.be (R.L.); 6 Center of Microbial and Plant Genetics, KU Leuven, 3000 Leuven, Belgium; vera.vannoort@kuleuven.be; 7 Department of Microbiology, Immunology and Transplantation, Allergy and Clinical Immunology Research Group, KU Leuven, 3000 Leuven, Belgium; 8 Pharmacy Department, University Hospitals Leuven, 3000 Leuven, Belgium; Isabel.spriet@uzleuven.be (I.S.); david.devolder@uzleuven.be (D.D.); 9 Department of Pharmaceutical and Pharmacological Sciences, Clinical Pharmacology and Pharmacotherapy, KU Leuven, 3000 Leuven, Belgium; 10 Department of Intensive Care Medicine, University Hospitals Leuven, 3000 Leuven, Belgium; yves.debaveye@uzleuven.be; 11 Department of Laboratory Medicine, University Hospitals Leuven, 3000 Leuven, Belgium; melissa.depypere@uzleuven.be; 12 Laboratory of Clinical Bacteriology and Mycology, Department of Microbiology, Immunology and Transplantation, KU Leuven, 3000 Leuven, Belgium; 13 Department of Pneumology, University Hospitals Leuven, 3000 Leuven, Belgium; lieven.dupont@uzleuven.be; 14 Department of Internal Medicine, University Hospitals Leuven, 3000 Leuven, Belgium; paul.demunter@uzleuven.be (P.D.M.); willy.peetermans@uzleuven.be (W.E.P.); 15 Laboratory for Clinical Infectious and Inflammatory Disorders, Department of Microbiology, Immunology and Transplantation, KU Leuven, 3000 Leuven, Belgium; 16 Institute of Biology, Leiden University, 2333 BE Leiden, The Netherlands; 17 Laboratory for Molecular and Cellular Technology, Queen Astrid Military Hospital, 1120 Brussels, Belgium; maia.merabishvili@mil.be (M.M.); jean-paul.pirnay@mil.be (J.-P.P.)

**Keywords:** bacteriophage therapy, difficult-to-treat infections, safety, efficacy, patient registry

## Abstract

In times where only a few novel antibiotics are to be expected, antimicrobial resistance remains an expanding global health threat. In case of chronic infections caused by therapy-resistant pathogens, physicians have limited therapeutic options, which are often associated with detrimental consequences for the patient. This has resulted in a renewed interest in alternative strategies, such as bacteriophage (phage) therapy. However, there are still important hurdles that currently impede the more widespread implementation of phage therapy in clinical practice. First, the limited number of good-quality case series and clinical trials have failed to show the optimal application protocol in terms of route of administration, frequency of administration, treatment duration and phage titer. Second, there is limited information on the systemic effects of phage therapy. Finally, in the past, phage therapy has been applied intuitively in terms of the selection of phages and their combination as parts of phage cocktails. This has led to an enormous heterogeneity in previously published studies, resulting in a lack of reliable safety and efficacy data for phage therapy. We hereby present a study protocol that addresses these scientific hurdles using a multidisciplinary approach, bringing together the experience of clinical, pharmaceutical and molecular microbiology experts.

## 1. Introduction

Each year, over 700,000 people die due to drug-resistant infections. Without policies to stop the growing spread of antimicrobial resistance, this alarming number has been predicted to increase up to 10 million deaths every year by 2050 [1]. Pathogens such as methicillin-resistant *Staphylococcus aureus* (MRSA) and multidrug-resistant *Pseudomonas aeruginosa* (MDR-PA) present a significant health threat [2]. Within the healthcare setting alone, for example, MRSA infections are currently estimated to affect over 150,000 patients annually in the European Union, resulting in additional in-hospital costs of 380 million euros for EU healthcare systems [3]. Therefore, the World Health Organization (WHO) advocates increasing research focused on antimicrobial resistance [4]. Nevertheless, there is relatively limited interest from pharmaceutical companies to engage in the process of antibiotic drug development, particularly considering the potential restrictions on the use of any new antibiotics and the risk of rapid resistance development [4]. In addition, some pathogens are able to form biofilms, which makes them inaccessible to the human immune system and intrinsically resistant to antibiotics [3]. Furthermore, the close proximity of bacteria in a biofilm environment facilitates the dissemination of antibiotic-resistance genes through horizontal gene transfer, thereby increasing its recalcitrance to antibiotic treatment [5,6]. Therefore, especially for (chronic) infections caused by (multidrug-) resistant strains, treatment options are limited. This often compels physicians to take therapeutic measures with an important impact on the patient, such as lifelong suppressive antibiotics or amputation of the affected limb. For the abovementioned reasons, scientists are urgently seeking alternatives for antibiotics. One of these strategies is bacteriophage (phage) therapy.

Despite the promising clinical outcomes described in previous reports, these clinical applications have revealed some important hurdles that need to be overcome. First, the limited number of well-documented case series and clinical trials have failed to show the optimal application protocol in terms of route of administration, frequency of administration, treatment duration and phage titer. Second, there is limited information on the systemic effects of phage therapy. Finally, in the past, phage therapy has been applied intuitively in terms of phage selection and their combination in phage cocktails. These critical knowledge gaps regarding the safety and efficacy of phage therapy have contributed to the fact that competent authorities are hesitant towards the more widespread implementation of phage therapy in Western clinical practice. In Belgium, the application of phage therapy has a regulatory framework as magistral preparations [7]. In this regard, our center has approved the application of phage therapy as standard-of-care only in patients for whom no curative treatment alternatives (antibiotic and/or surgical) are available (‘last-resort cases’). A multidisciplinary phage task force, referred to as the Coordination group for Bacteriophage therapy Leuven (CBL), was therefore set up. Using this multidisciplinary approach, the first treatment successes in our center were already reported [8]. The CBL screens patients with difficult-to-treat infections, evaluates who could benefit from phage therapy and sets up the treatment protocol. With this study, the CBL aims to gain insight in the safety and efficacy of phage therapy by integrating and optimizing phage therapy in three distinct medical disciplines (with three distinct routes of administration), facilitating long-term follow-up of patients. We acknowledge that in this target population, where the infection will already be at an advanced stage, timing will be of the essence. With this study and the safety data it will generate, we hope to be able to broaden the indications for phage therapy in the future.

## 2. Materials and Methods

### 2.1. Study Objectives

The main objective of the study is to collect the safety and efficacy data of patients diagnosed with a difficult-to-treat musculoskeletal infection (MSI), chronic rhinosinusitis (CRS) or sepsis who have been treated with phage therapy. Specifically, this study aims at:

- Implementing phage therapy in three medical disciplines and creating a prospective patient registry;
- Gaining insight in the kinetics of phage therapy using three different routes of administration;
- Characterizing the interaction between phage and bacteria and optimizing future phage cocktails by applying a genome-based approach.

### 2.2. Study Design

This is a prospective, monocentric and observational registry. In consultation with the hospital’s Ethics Committee, phage therapy is considered to be standard-of-care only in patients with difficult-to-treat infections for whom curative treatment alternatives (antibiotic and/or surgical) are not available (‘last-resort cases’). Whether the patient will receive phage therapy (phage treatment group) or a standardized non-curative (e.g., amputation, chronic antibiotic suppression) treatment (control group) depends solely on the susceptibility of the causative pathogen(s) against the available phages. More specifically, in case active phages are not available against all causative pathogen(s), the patient will not undergo phage therapy.

### 2.3. Study Population

The selection of infectious indications for phage therapy in this study protocol is based on the clinical expertise of a multidisciplinary team. Moreover, these indications represent different levels of complexity, from intraoperative and local (MSI) to topical, intranasal (CRS) and systemic, intravenous (sepsis) applications.

#### 2.3.1. Sample Size Calculation

Since data on the application of phage therapy for MSI, CRS and sepsis are currently scarce, we are unable to perform a power analysis due to the absence of an objective effect size. The main aim of this registry is to obtain safety and efficacy data. Based on previously received requests for phage therapy, an estimated 150 patients will be included. Furthermore, within this patient population, approximately 75 will have a positive phagogram and can therefore be treated with phage therapy.

#### 2.3.2. Eligibility

**Figure 1:**
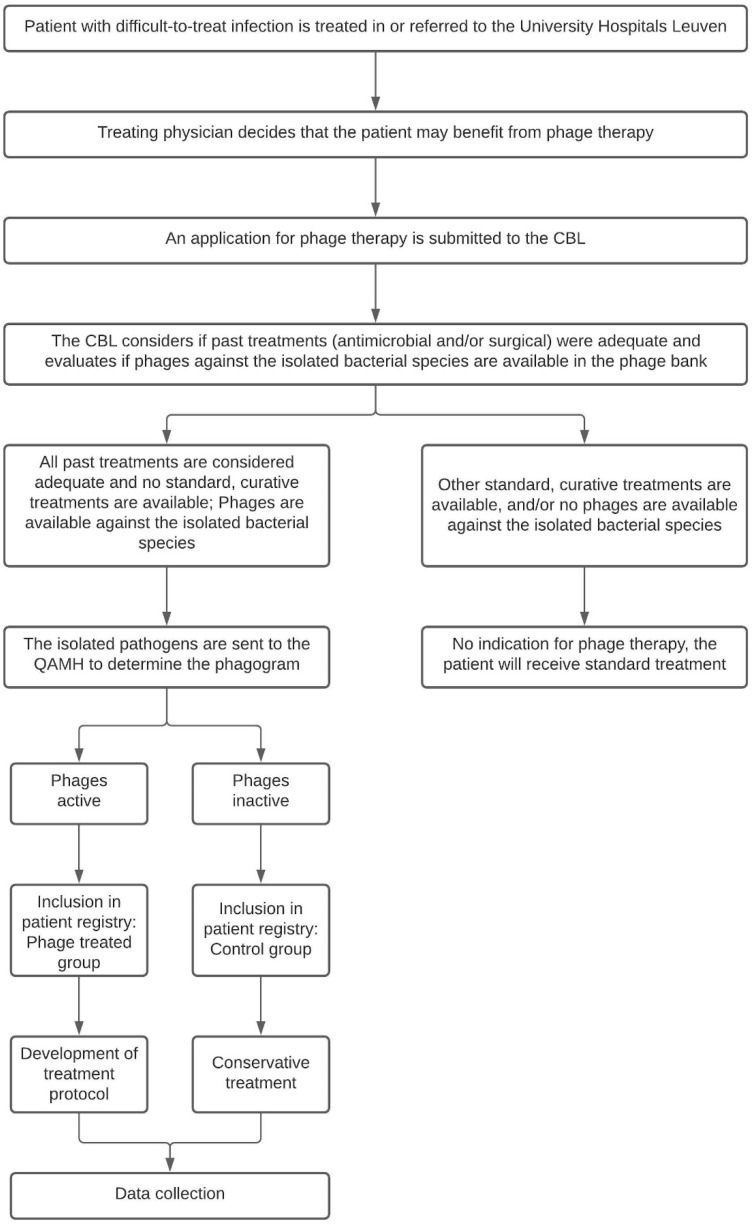
Flowchart of the PHAGEFORCE study. Patients are screened for eligibility by the CBL. Based on the availability of active phages, the patient can (phage treatment group) or cannot (control group) be treated with phage therapy. Regardless of which group the patient is in, all data related to the infection will be collected. CBL: Coordination group for Bacteriophage therapy Leuven; QAMH: Queen Astrid Military Hospital.

When the treating physician presumes his or her patient may benefit from phage therapy, the patient is presented to the CBL. The CBL will look into the patient’s medical history and determine if past treatments were adequate and if there are any other curative (surgical or antibiotic) treatment options available. In most cases, the causative pathogen is known at the time of presentation to the CBL. They will therefore also evaluate if phages are available in the phage bank against the previously isolated bacterial species. If that is the case and if no alternative curative treatments are available, the patient is eligible for inclusion in the PHAGEFORCE study. The study flowchart including the aforementioned application procedure is presented in Figure 1.

#### 2.3.3. Inclusion Criteria

All patients:

- Diagnosed with a difficult-to-treat MSI, CRS or sepsis, and;
- For whom all previous treatments (surgical and antibiotic) have failed or for whom no other curative treatment options are available (i.e., ‘last resort cases’, based on the CBL assessment), for example in case of bacterial resistance, and;
- For whom phages targeting the isolated bacterial species are available in the phage bank, and;
- Who have given informed consent to have their data collected in a patient registry.

#### 2.3.4. Exclusion Criteria

All patients:

- With an infectious disease other than MSI, CRS or sepsis, and/or;
- For whom standard curative treatment alternatives are still available, and/or;
- For whom phages targeting the isolated bacterial species are not available in the phage bank, and/or;
- Who refuse to give their informed consent.

### 2.4. Study Procedures

After the CBL has deemed the patient eligible for phage therapy, informed consent will be asked from the patient, either at the outpatient clinic or at the hospital ward. The informed consent covers prospective data collection prior to, during and after (phage) treatment. Study-specific variables that will be collected for each indication are listed below.

#### 2.4.1. Baseline Parameters

When informed consent is obtained, parameters regarding the patient’s demographics, medical history and comorbidities, as well as details on the current infection are collected. Patient demographics comprise variables including the patient’s age, sex, weight, height and ethnicity. The medical history includes pre-existing conditions (e.g., cardiovascular diseases, asthma, diabetes). Any concomitant medications associated with these pre-existing conditions will be recorded. The American Society of Anesthesiologists (ASA) score for MSI patients and the baseline Lund–Kennedy (based on nasal endoscopy), Modified Davos (based on nasal endoscopy) and Lund–Mackay (based on CT evaluation) scores for CRS patients will be recorded. For sepsis patients, the baseline Sequential Organ Failure Assessment (SOFA) score will be collected. Radiographic images and/or clinical pictures are taken as part of the standard-of-care.

**Figure 2:**
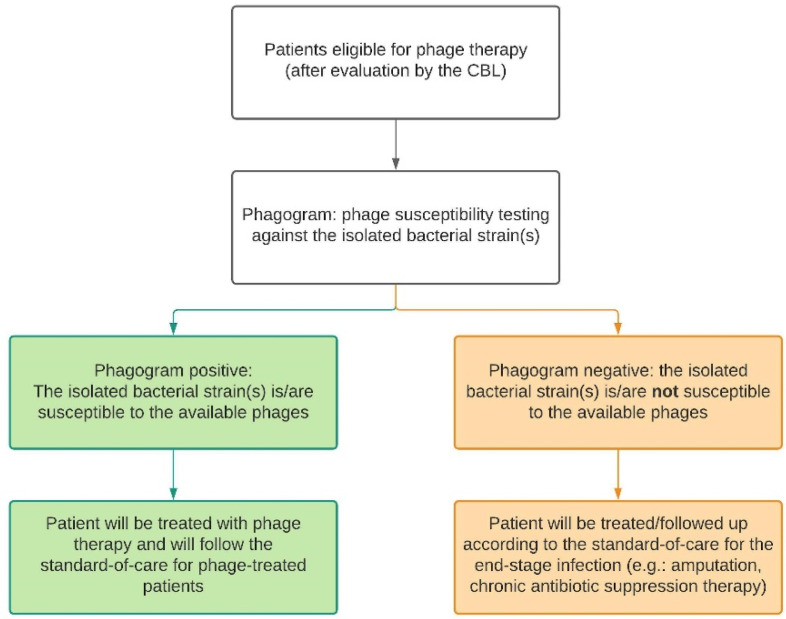
Depending on the result of the phagogram, the patient will follow either the phage therapy trajectory (green) or the control trajectory (orange). For each trajectory, there is a standard-of-care protocol. CBL: Coordination group for Bacteriophage therapy Leuven.

If no recent cultures are available for susceptibility testing, bacteriological samples will be taken and cultured. For MSI patients, this includes intraoperative deep tissue cultures taken during a surgical debridement. Nasal swabs and hemocultures will be taken from CRS and sepsis patients, respectively. The isolated pathogens will be sent to the Queen Astrid Military Hospital (QAMH) for susceptibility testing against phages in their phage bank. Depending on the susceptibility of the isolates, also referred to as the ‘phagogram’, the patient can be included in the phage treatment group or the control group where no alternative curative treatment options are available. In case of polymicrobial infections, all isolated bacterial species have to be susceptible to the available phages. Furthermore, the CBL will set up the treatment plan accordingly and document this in the patient’s medical file. Therefore, in this study, there are two types of standard-of-care, as displayed in Figure 2 and defined below.

#### 2.4.2. Treatment-Specific Parameters: Phage Treatment Group

If all eligibility criteria are met and a positive phagogram is obtained, the patient will be included in the phage treatment group. The CBL will design the treatment protocol and document it in the CBL final treatment plan, which is also registered in the patient’s medical file. Phages will be administered locally, through a draining system for MSI patients (as previously described [8]), intranasal for CRS patients and intravenously for sepsis patients. The phages that will be used are produced according to a monograph at the QAMH [7]. Furthermore, these phages are well-characterized, in that the genetic sequence and therefore the confirmation of each phage’s strictly lytic profile and absence of undesired genetic determinants (e.g., toxins and antibiotic-resistance genes) are available. These analyses are verified independently by Sciensano, the Belgian federal research institute for public health, and summarized in a so-called genetic passport. Moreover, each batch of phages (phage stock) produced at the QAMH is quality controlled by Sciensano before it can be used in humans [7,8]. Furthermore, phage stock titers will be determined frequently and prior to each dilution for phage therapy. For each individual phage that will be used, stability tests of the diluted preparations will also be performed for the different indications. In this way, we know how frequently we need to prepare new diluted preparations during the treatment to maintain a therapeutic target titer.

Improvements in pharmacological insights during phage therapy development is critical, particularly towards obtaining a better understanding of treatment failures [9]. As it is likely that the systemic exposure of a virulent phage is significantly modified due to its adsorption to the susceptible bacterial cells causing the infection, it becomes difficult to extrapolate the systemic exposure of other antimicrobial drugs to that of the phages. Furthermore, virulent phages multiply within their host and, as a result, while classic antibiotics decay over time, bacteriophage titers increase in the presence of a susceptible host [10]. Multiple samples will be taken (as detailed in Table S1) to monitor the patient and treatment progress. These samples will also be used to gain insight into the physiology of the phages during phage therapy, as detailed below.

##### Phage Dynamics

- Assessment of Safety

One of the principal aims of this study is to evaluate the safety profile of the applied phage therapy protocols. Patients treated with phage therapy are closely monitored during phage therapy. Their clinical parameters (e.g., heart rate, blood pressure, oxygen level, temperature, respiratory rate) will be collected as detailed in Table S1 and stored in the patient registry. In addition, it is a standard-of-care to have several blood tests and samples analyzed, as listed in Table S1, to monitor the general health status of the patients. The following (blood) laboratory tests are required for monitoring (and optimization) of the phage therapy regimen and are therefore considered standard-of-care for patients treated with phage therapy:

- Complete blood count (CBC);
- Basic metabolic panel;
- Inflammatory parameters;
- Lactic acid, Creatine kinase;
- Liver function tests;
- Antiphage antibodies (serum);
- Phage titration and isolation from draining fluid (MSI), nasal swabs (CRS) or serum (sepsis).

Even though no phage-related toxicity has been described or is to be expected, monitoring these parameters closely ensures that quick action can be undertaken in case of abnormalities. In this study, only adverse events or complications directly related to the condition or treatment of the infection will be collected. Specifically, for sepsis patients, only those safety parameters which could theoretically be related to the administration of phage therapy will be recorded in the patient’s digital medical record and in the patient registry. Reporting all untoward medical events on the intensive care unit (ICU) would not be practically feasible nor relevant, given the heterogeneity of the population and its inherent susceptibility to various medical events and complications. In case an adverse event (AE) or serious adverse event (SAE) occurs during the study period, this will be documented in the registry. The investigators and study personnel will seek information on AEs during each patient contact until three months after the final phage administration. All events that can be related to the infection or treatment of infection, whether reported by the patient or noted by the study personnel, will be recorded in the patient’s medical record and in the patient registry within a reasonable time after becoming aware. If available, the diagnosis will be reported on the AE page, rather than the individual signs or symptoms. If no diagnosis is available, the investigator will record each sign and symptom as individual AEs. Each AE will be followed up until resolved or until the end of the patient’s study participation, whichever occurs first. An independent data safety monitoring board (DSMB) is also set up for this study to help monitor the safety of phage therapy in our center. The DSMB consists of infectious disease physicians, intensive care specialists, pneumologists, clinical pharmacists, microbiologists and surgeons. For every 25 patients who are included, a report will be provided to the DSMB. They will go over any possible AEs, evaluate the overall outcome and provide feedback to the CBL.

- Assessment of Efficacy

To assess the potential of phage therapy with respect to infection eradication, a combination of quantitative and qualitative outcome measures will be evaluated. As stated above, blood laboratory test results and clinical parameters will be collected to monitor the evaluation of the patient’s health status during treatment. The bacterial load during and after phage therapy will be measured. Several bacterial cultures will be taken from all patients. For CRS patients this entails nasal swabs, for sepsis patients this includes hemocultures. In case of MSI, it is not feasible to have multiple deep tissue cultures taken after the wound has been closed. As an alternative, during phage therapy, fluid collected in the draining system that is used for the application of phage therapy will be cultured. As these draining tubes are typically removed at the end of phage therapy, cultures can no longer be obtained during the follow-up period, unless there is a need for revision surgery (i.e., in case of recurrence of infection). Therefore, the main outcome measure for MSI patients is a disease-free period of at least one year after cessation of therapy. The bacteria and phages that are isolated from the various samples described above and listed in Table S1, will be stored for further analysis at −80 °C and 4 °C, respectively (see Section 2.5. Translational Research).

For CRS patients, specific clinical and quantitative outcome measures are available. These include endoscopic (Modified Davos, Lund–Kennedy), radiological (Lund–Mackay) and combined (European Position Paper on Rhinosinusitis and Nasal Polyps (EPOS) criteria for controlled or uncontrolled CRS) scoring systems, which will be collected and compared before, during and after phage therapy. Moreover, to evaluate the olfactory function in CRS patients, smell tests (Sniffin’ Stick test) will be carried out before and after phage therapy. For sepsis patients, the SOFA score will be recorded before, during and after treatment. The SOFA score is an internationally used score to objectively determine the degree of multiple organ failure in critically ill patients. A (rapid) decrease of the SOFA score (delta SOFA) is associated with a better survival and vice versa. Delta SOFA is defined as the difference between the SOFA score at baseline (prior to phage therapy) and the SOFA score at the final day of phage therapy.

Regarding qualitative outcome measures, patient-reported outcomes (PROs) will be collected from CRS and MSI patients. These include the PROMIS (patient-reported outcomes measurement information system) on global health and pain interference (for both CRS and MSI patients), PROMIS on physical function (for MSI patients) and the Sino-Nasal Outcome Test-22 (SNOT-22) and Visual Analogue Scale (VAS) for CRS patients.

##### Phage Kinetics

To evaluate the local phage titer during phage therapy, the draining fluid of MSI patients and nasal swabs from CRS patients will be collected. To evaluate the systemic exposure of phages, phage titration will be performed on the collected serum samples. In all blood samples, phage quantification will be performed using the double agar overlay method (viable fraction) and qPCR (total titer in the blood). Viable phages will be isolated and their whole genome sequenced to identify possible mutations (see Translational research). Serum samples taken from sepsis patients will help elucidate how long phages remain detectable in the blood before the next intravenous administration. This information will be crucial to evaluate the currently used application protocol. Furthermore, based on hemoculture results, we will evaluate phage persistence after cessation of therapy, in the presence or absence of their bacterial host. The bacterial load in the blood will be quantified by determining and extrapolating ‘time-to-positivity’ (TTP) of the hemocultures. The TTP is defined as the time from the start of incubation to a positive signal.

The clearance of phages by the immune system may affect the efficacy of phage therapy [11]. Low titers of phage-specific antibodies are common in patients because phages are encountered on a daily basis, but titers may increase during phage therapy. The induction of the innate immune system (clearing phages through phagocytosis (i.e., the reticuloendothelial) system) as well as the adaptive immune system (production of phage-neutralizing antibodies), has been associated with early depletion of phages and subsequent impairment of efficacy [12,13]. It may be necessary to compensate for this phenomenon by repeating phage administration, increasing phage titer, using different phages or a phage cocktail. Therefore, from all patients who are treated with phage therapy, serum samples will be processed using the phage neutralization assay, as previously described [8], to detect antiphage antibodies on the time points listed in Table S1.

#### 2.4.3. Follow-Up Parameters: Phage Treatment Group

Patients who were treated with phage therapy will follow the standard-of-care for phage therapy until three months after the final administration of phage therapy. After these three months, they will follow the standard-of-care follow-up schedules for the underlying infection, confer patients in the control group. Data will be collected until one year after the final phage administration.

#### 2.4.4. Treatment-Specific and Follow-Up Parameters: Control/Standard Treatment Group

The CBL will also set up the treatment protocol for control patients (standard, non-curative treatment). Control patients will only be subjected to blood analyses and sampling when required according to the standard-of-care for the underlying pathology. The results of all blood and culture tests that are performed in that period with respect to the underlying infection will be recorded in the patient registry. All bacteria isolated from the infected site will be stored for further analysis. All eligible MSI and CRS patients will also be asked to fill out PROMIS (for MSI and CRS) and SNOT-22 and VAS (CRS) questionnaires at standard follow-up outpatient visits. Patients will be followed, and the data will be collected until one year after the CBL’s final treatment plan.

### 2.5. Translational Research

#### 2.5.1. Microbiological Analysis of Patient Bacterial Isolates and Identification of Novel or Long-Circulating Phage Mutants

When different phages are available for treatment (based on the phagogram), phage compatibility will be assessed by investigating the interaction between phages inside the cocktail. The synergy between phages can be investigated, as described by Schmerer [14]. When phages are not available (based on a negative phagogram), the patient will be included in the control group, but the patient’s bacterial isolates can be used to isolate additional phages (e.g., from hospital sewage) to extend the phage bank for future patients. Alternatively, these phage-resistant bacteria can also be used to train phages to broaden their bacterial host range, which is valuable in order to comprise the full diversity of the pathogens and to surpass bacterial-resistance development [15]. The creation of such host range mutants will be performed according to a modified Appelmans’ protocol [16].

All bacterial isolates taken during and after phage treatment will be tested for sensitivity against the applied phages and the efficiency of plating (EOP) will be compared to the initial (pre-treatment) one. The EOP is defined as the number of plaques a phage is able to produce when incubated with a bacterial strain relative to the number of plaques the phage is able to produce when incubated with its host or reference bacterial strain. Antibiograms will be compared to the baseline antibiogram to determine if the susceptibility to antibiotics has changed and look for synergistic interactions [17]. Furthermore, bacterial isolates will be fully characterized by whole genome sequencing, using a combination of Illumina and Nanopore sequencing [18] to obtain optimal genome assemblies. This will be crucial to improve the functional annotation of the genomes. We will focus on the annotation of elements that could be directly linked to lytic phage efficacy. Genome features, such as plasmids, CRISPR–Cas systems, prophages, restriction–modification (R-M) systems and receptors are all linked to susceptibility [19].

During and after phage treatment, phage mutants that have naturally evolved to be more efficient in the patient environment, could also arise. Therefore, phage isolation from patient samples will be performed during and after treatment. These ‘in patient-optimized’ phages will then be characterized by whole genome sequencing and will be the basis for next-generation therapeutic phages that could be used in personalized phage therapy.

#### 2.5.2. Characterization of the Phage Library and Using Machine Learning to Predict Host Range Phenotype from the Genotype

By expanding our dataset with the clinical data from this study and in vivo-occurring resistance mechanisms, we aim to develop predictive models of phage infectivity. These models will enable us to set up a decision tool to improve phage cocktail design and further fundamental insights into the determinants of phage infectivity. As previously mentioned, available phages will be tested for phage infectivity on each bacterial isolate (before treatment). These data will not only be used to identify the phages for treatment, but also to enhance our dataset for the machine learning approaches to predict phage infectivity based on whole genome data. We want to capitalize on the large amounts of sequencing data that will be used to train a decision algorithm to better predict infectivity towards a new bacterial host. As the number of genomics features that could be employed is vast, feature selection will be performed based on the presence of genetic systems linked to phage susceptibility, including prophages, CRISPR–Cas systems, R-M or known genes coding for membrane receptors.

### 2.6. Statistical Analysis

As the aim of this registry is mainly descriptive and exploratory, patient characteristics and outcomes recorded at standard-of-care scheduled follow-up assessments will be presented using simple summary statistics.

Categorical variables will be summarized using the frequency and percentage for each category. Continuous variables will be summarized using the mean, standard deviation, inter-quartile range and minimum and maximum values. These summary statistics will in addition be presented according to the clinically relevant categories; i.e., according to the treatment received.

Complications will be reported both at the patient and the AE level. Given the exploratory nature of this study, any results will have to be interpreted carefully.

### 2.7. Ethics and Regulatory Approvals

The study will be conducted in compliance with the principles of the Declaration of Helsinki (2013), the principles of Good Clinical Practice (GCP) and will be in accordance with all applicable regulatory requirements. This protocol and related documents were approved by the Ethics Committee Research UZ/KU Leuven (S64854), in consultation with the Chief Medical Officer and medical board of the University Hospitals Leuven.

The study team shall treat all information and data related to the study as confidential and shall not disclose such information to any third parties or use such information for any purpose other than the performance of the study. The collection, processing and disclosure of personal data, such as patient health and medical information, are subject to compliance with applicable personal data protection and the processing of personal data (General Data Protection Regulation (EU 2016/679)).

### 2.8. Data Handling and Management

Data will be submitted to an electronic Case Report Form (eCRF). Patient data are coded, implying there is a link between the data and the individual who provided it. The subject’s name or other identifiers will be stored separately from their research data and replaced with a unique code to create a new identifier for the subject. The research team is obliged to protect the data from disclosure outside the research according to the terms of the research protocol and the informed consent document.

For this registry-based study, an eCRF will be designed using RedCap to accommodate all study-specific features. Access to the eCRF is password protected and specific functions are assigned (e.g., study coordinator, investigator, monitor). The eCRF is completed in a timely manner after a patient’s visit.

## 3. Discussion

To date, competent authorities in most European countries remain hesitant to implement phage therapy in clinical practice. Despite an increasing number of publications on the topic, this is mainly due to a lack of high-quality safety and efficacy data. Recently, a systematic review regarding the safety and efficacy of phage therapy was performed by our consortium [20]. Although a low rate of adverse events was reported in the included studies, there was a serious discrepancy in the reporting of adverse events between different case studies and even between randomized controlled trials. Furthermore, not all studies described how and if the AEs were collected. In the same systematic review, high efficacy rates were described for all medical disciplines; however, the evidence quality was scored as low to moderate—according to the GRADE (Grading of Recommendations Assessment, Development and Evaluation) approach—due to a heterogeneity between studies. Examples of heterogeneity are different study designs, the use of different administration protocols and the application of personalized phage therapy compared to the administration of fixed phage cocktails [20].

For the abovementioned reasons, the PHAGEFORCE study protocol was developed. To our knowledge, this is the first attempt to set up a standardized, multidisciplinary approach to implement phage therapy in three distinct medical disciplines. By collecting data in a standardized manner, using a patient registry, this study will help evaluate the safety profile of the applied phage therapy protocols for three different routes of administration (i.e., local, topical and intravenous). Preliminary efficacy data will be generated, which can give rise to more insight into the optimal treatment protocol. The creation of this patient registry, which collects information in a standardized way, is an ideal method to characterize the course of the infection, to observe treatment effects and variations in outcome and to identify (risk) factors that correlate with a specific outcome and quality-of-life. Furthermore, our registry is set up in such a way that it will be possible in the future for other centers to join or to combine datasets. The samples that are collected and analyzed will also lead to more insight into the physiology of phage therapy when applied locally, intranasal or intravenously. Further expansion of our phage bank with compatible and ‘in patient-optimized’ phages will be crucial as isolate susceptibility is still a key factor to exclude patients from therapy. A positive feedback loop is generated by embracing longer-circulating phages and observing the consequences of resistance development against phages. This, in combination with co-evolution and machine learning modelling to optimize phage selection and cocktail design, provides a new and integrated approach that has not yet been explored. Furthermore, based on genomic features, we anticipate that novel mechanisms of phage–bacteria interaction will emerge that can trigger further systems biology research.

## 4. Conclusions

In conclusion, for the first time, the PHAGEFORCE study will enable us to evaluate the safety profile of the applied phage therapy protocols for three different routes of administration. Together with the efficacy data that will be collected, this can act as the foundation for future clinical studies. From a molecular microbiology perspective, this study will help us gain insight into phage–bacteria interactions and optimize future phage selection and cocktail design.

## Supplementary Materials

The following are available online at https://www.mdpi.com/article/10.3390/v13081543/s1, Table S1: Flowchart of tests and sampling performed in patients receiving phage therapy.
