# Supplementary material for: Bacteriophage Therapy for Difficult-to-Treat Infections: The Implementation of a Multidisciplinary Phage Task Force (The PHAGEFORCE Study Protocol)
Source: Viruses. 2021 Aug 5;13(8):1543. doi: 10.3390/v13081543 (PMC8402896; doi:10.3390/v13081543)
Supplement: Supplementary file 1 [file viruses-13-01543-s001.zip › List of abbreviations.pdf]

List of abbreviations

AE	Adverse event
ASA	American Society of Anesthesiologists
Cas	CRISPR-associated system
CBC	Complete blood count
CBL	Coordination group for Bacteriophage therapy Leuven
CRISPR	Clustered Regularly Interspaced Short Palindromic Repeats
CRS	Chronic rhinosinusitis
DSMB	Data Safety Monitoring Board
eCRF	Electronic case report form
EOP	Efficiency of plating
EPOS	European Position Paper on Rhinosinusitis and Nasal Polyps
EU	European Union
GCP	Good clinical practice
GRADE	Grading of Recommendations Assessment, Development and Evaluation
ICU	Intensive Care Unit
MDR-PA	Multidrug-resistant *Pseudomonas aeruginosa*
MRSA	Methicillin-resistant *Staphylococcus aureus*
MSI	Musculoskeletal infection
P. aeruginosa	*Pseudomonas aeruginosa*
PRO	Patient-reported outcomes
PROMIS	Patient-reported outcomes measurement information system
QAMH	Queen Astrid Military Hospital
qPCR	Quantitative polymerase chain reaction
R-M	Restriction-modification
S. aureus	*Staphylococcus aureus*
SAE	Serious adverse event
SNOT-22	Sino-nasal outcome test-22
SOFA	Sequential Organ Failure Assessment
TTP	Time-to-positivity
VAS	Visual Analogue Scale
WHO	World Health Organisation
