# Supplementary material for: Bacteriophage Therapy for Difficult-to-Treat Infections: The Implementation of a Multidisciplinary Phage Task Force (The PHAGEFORCE Study Protocol)
Source: Viruses. 2021 Aug 5;13(8):1543. doi: 10.3390/v13081543 (PMC8402896; doi:10.3390/v13081543)
Supplement: Supplementary file 1 [file viruses-13-01543-s001.zip › Study Protocol_PHAGEFORCE.pdf]

Protocol Full Title prospective observational trial:

Bacteriophage therapy for difficult-to-treat infections: the implementation of a multidisciplinary phage task force

Protocol Acronym/short title:

PHAGEFORCE

Version and date of final protocol:

Version 2, 7 January 2021

Sponsor:

Name: UZLeuven
Address: Herestraat 49, 3000 Leuven

Principal Investigator:

Name: Willem-Jan Metsemakers
Address: Department of Trauma Surgery, University Hospitals Leuven, Herestraat 49, 3000 Leuven
Telephone: 016 34 13 25
Email: willem-jan.metsemakers@uzleuven.be

Sub-investigators:

Name: Jolien Onsea
Address: Department of Trauma Surgery, University Hospitals Leuven, Herestraat 49, 3000 Leuven
Telephone: 016 34 20 41
Email: jolien.onsea@uzleuven.be

Name: Laura Van Gerven
Address: Department of otorhinolaryngology, University Hospitals Leuven, Herestraat 49, 3000 Leuven
Telephone: 016 33 63 90
Email: laura.vangerven@uzleuven.be

Name: Saartje Uyttebroek
Address: Department of otorhinolaryngology, University Hospitals Leuven, Herestraat 49, 3000 Leuven
Telephone: 016 33 63 79
Email: Saartje.uyttebroek@uzleuven.be

Name: Yves Debaveye
Address: Department of intensive care medicine, University Hospitals Leuven, Herestraat 49, 3000 Leuven
Telephone: 016 34 03 94
Email: yves.debaveye@uzleuven.be

Name: Isabel Spriet
Address: Clinical pharmacy, University Hospitals Leuven, Herestraat 49, 3000 Leuven
Telephone: 016 34 12 61
Email: isabel.spriet@uzleuven.be

Name: Melissa Depypere
Address: Department of Laboratory Medicine, University Hospitals Leuven, Herestraat 49, 3000 Leuven
Telephone: 016 34 50 31
Email: Melissa.depypere@uzleuven.be

Name: Paul De Munter
Address: Department of General Internal Medicine, University Hospitals Leuven, Herestraat 49, 3000 Leuven
Telephone: 016 34 09 12
Email: paul.demunter@uzleuven.be

Name: Willy Peetermans
Address: Department of General Internal Medicine, University Hospitals Leuven, Herestraat 49, 3000 Leuven
Telephone: 016 34 02 27
Email: willy.peetermans@uzleuven.be

Name: Lieven Dupont
Address: Department of Respiratory Diseases, University Hospitals Leuven, Herestraat 49, 3000 Leuven
Telephone: 016 34 68 16
Email: lieven.dupont@uzleuven.be

Signatures

______________________________ ________________________

Principal Investigator Date

Print Name:

______________________________ ________________________

Date

Print Name:

Table of Contents

1. Study Synopsis 5
2. Background and rationale 7
3. Trial objectives and Design 7
   3.1 Trial objectives 7
   3.2 Primary endpoints 7
   3.3 Secondary endpoints 7
   3.4 Trial Design 7
   3.5 Study diagram 7
   3.6 Trial Flowchart 7
4. Selection and withdrawal of subjects 8
   4.1 Inclusion criteria 8
   4.2 Exclusion criteria 8
   4.3 Expected duration of trial 8
5. Trial Procedures 8
   5.1 By visit 8
   5.2 Laboratory tests 9
   5.3 Other investigations 9
6. Assessment of efficacy 9
7. Assessment of Safety 9
   7.1 Specification, timing and recording of safety parameters 9
   7.2 Procedures for recording and reporting adverse events 9
   7.3 Treatment stopping rules 9
8. Statistics 9
   8.1 Sample size 9
   8.2 Analysis 10
9. Quality assurance 10
10. Direct access to source data and documents 10
11. Ethics and regulatory approvals 10
12. Data Handling 11
13. Data Management 11
14. Translational research 11

15. Publication Policy 11
16. Insurance/Indemnity 12
17. Financial Aspects 12
18. References 12

1. Study Synopsis

Title of clinical trial	Bacteriophage therapy for difficult-to-treat infections: the implementation of a multidisciplinary phage task force
Protocol Short Title/Acronym	PHAGEFORCE
Sponsor name	UZ Leuven
Principal Investigator	Prof. dr. Willem-Jan Metsemakers
Medical condition or disease under investigation	Last resort musculoskeletal infections, sepsis and chronic rhinosinusitis
Purpose of clinical trial	To understand the physiology of phage therapy using three different routes of administration.
Primary objective	To gain insight in the safety and tolerability of phage therapy for each route of administration
Secondary objective (s)	To gain insight in the efficacy of phage therapy for each indication
Trial Design	Prospective, non-interventional
Sample Size	N/A
Summary of eligibility criteria	All patients with a musculoskeletal infection or sepsis or chronic rhinosinusitis for whom all previous treatments (antibiotic/surgical) have failed or are likely to fail, or for whom no other treatments are available (i.e. in case of antibiotic resistance).
Maximum duration of study for a Subject	The patient will be followed for a year after the final phage administration (phage treated patients) or after the final decision of the MPTF after having received the phagogram results (control patients).
Version and date of final protocol	Version 2, 7 January 2021
Version and date of protocol amendments	n/a

2. Background and rationale

The threat of antimicrobial resistance

Antimicrobial resistance (AMR) is one of the biggest threats to global health today, as stated by the

World Health Organization (WHO) [1]. Pathogens such as methicillin-resistant *Staphylococcus aureus* (MRSA) and multidrug-resistant *Pseudomonas aeruginosa* (MDR-PA) present a significant health threat. Within the healthcare setting alone, for example, MRSA infections are estimated to affect over 150.000 patients annually in the European Union (EU), resulting in additional in-hospital costs of EUR 380 million for EU healthcare systems [2]. The WHO advocates increasing research and development on AMR. Although some new antibiotics are in the pipeline, the numbers are insufficient to address the needs, and the financial return-on-investment discourages further development, particularly considering the potential restrictions on the use of any new antibiotics and the risk of rapid resistance development [1]. Furthermore, some pathogens are able to form biofilms on medical devices, making them inaccessible to the human immune system and antibiotics, without even requiring antibiotic resistance genes. Therefore, especially for (chronic) infections caused by (multi-drug) resistant strains, treatment options are limited. This often compels physicians to take measures that have an important impact on the patient (e.g. lifelong suppressive antibiotics, amputation of the affected limb). Scientists are therefore urgently seeking antimicrobial alternatives and one of these is bacteriophage therapy.

The potential of phage therapy to treat chronic infections

(Bacterio)phages are the natural enemies of bacteria. Strictly lytic phages recognize and infect their target bacteria, converting the cell into a 'phage-producing machine'. At the end of the infection cycle, progeny phages are released and the host bacteria destroyed. From these basic biological steps, it is widely recognized that phages have several important traits that contribute to their therapeutic potential. First of all, phages **self-amplify**, which is a large asset that contributes to their efficacy and distinguishes them from conventional antimicrobials [3]. Second, some phages display **polysaccharide depolymerases** on their tail structures, which can act as an adjuvant to phage infection by degrading the extracellular matrix of biofilm-associated bacteria [4]. Third, phages are considered to be safe as human tissue and normal human bacterial flora are not negatively affected, which can be attributed to their **high specificity** [5,6,3]. Finally, their modes of action tend not to be affected by bacterial antibiotic resistance mechanisms [7]. Within Europe, Belgian (and KULeuven) researchers are at the forefront of the implementation of phage therapy in clinical practice. In the past years, we have overcome key obstacles in establishing safe phage cocktails, optimized formulation and production and tackled legislative and patenting hurdles, leading to the first treatment successes [8-10]. Nevertheless, these first clinical applications have laid bare new hurdles associated with clinical implementation, which will be directly addressed in the aims of this study protocol [10].

Exploring the phage-bacteria interaction using machine learning

A key element in the successful application of phage therapy is the interaction between phages and their host. Generally, phage treatment protocols rely on historical practices from when phage therapy was primarily used in the former Soviet Union. While this decade-long experience in establishing cocktails is valuable, it also represents a black-box which limits development. To gain microbiological insights into phage susceptibility, gene knock-out studies classically provide insight in the relation between gene function and phenotype. However, this presents a huge effort with thousands of genes to be investigated. The current research of the Van Noort & Lavigne groups aspires to combine high-throughput sequencing and machine learning to gain novel insights into this infection process. This is possible through the following steps. (1) With whole genome sequencing, it has become possible to sequence large numbers of strains and map genomic feature to phenotypes. Next, variation between bacterial strains is established through the presence or absence of genes. In this regard, the core genome is defined as the set of genes that is shared between all strains of one species, whereas the

accessory genome is defined as the set of genes that is present in only one or a subset of strains. In the PhD of Cedric Lood, we have already established a unique library of over 500 sequenced clinical *P. aeruginosa* isolates and assessed phage infectivity of our entire phage library under *in vitro* conditions. (2) Machine learning approaches allow us to relate phenotypes (i.e. phage infectivity) to the binary features of the accessory genomes. The challenge lies in the large number of features (1'000s), where manual observations and standard statistical models tend to fail. We have established machine learning methods, being Support Vector Machines and Random Forests, and have established that phage susceptibility is predictable based on the gene content of both phages and bacteria.

From Random Forests, feature importance can be extracted and can be used to identify genes that are important for phage susceptibility. This fundamental research, which has provided an extensive and reliable dataset, serves as an essential basis to establish an informed strategy to establish patient-tailored phage cocktails.

Clinical application of phage therapy: standardized application protocol

In Belgium, applying phage therapy for difficult-to-treat (DTT) infections within the framework of magistral preparations is already approved [9]. In this regard, UZ Leuven decided to set up a Multidisciplinary Phage Task Force (MPTF), later referred to as the Coordination group for Bacteriophage therapy Leuven (CBL), consisting of Infectious Disease (ID) physicians, pneumologists, clinical pharmacists, microbiologists, surgeons and phage scientists. This group evaluates and selects patients who may benefit from phage therapy. Patients are eligible when they are diagnosed with one of the following infections for which antimicrobial and/or surgical treatments have failed or are not available (i.e., last resort cases): musculoskeletal infections, chronic rhinosinusitis or sepsis. Upon eligibility, and in collaboration with the Queen Astrid Military Hospital (QAMH), the isolated pathogens are tested against the available phage panel. If the phages are active, phage therapy can be planned, in consultation with the group. They ensure that the application protocol (route of administration, dose, frequency of administration/application, duration) in the respective patient populations is standardized.

Selection of medical fields for phage therapy

The selection of the above-mentioned infectious indications for phage therapy is based on clinical expertise (mainly MSI) and because these indications represent different levels of complexity, from topical (CRS) to intraoperative, local (MSI) and systemic applications (sepsis).

Musculoskeletal infections (MSI):

Although the rate of infectious complications after elective orthopaedic surgery remains low (~3%) [11], the incidence continues to rise not only due to the annual increase of elective joint replacement surgeries [12] but also due to an increase in the number of operatively treated fractures [13]. Moreover, the overall infection rate in musculoskeletal trauma remains high and can rise up to 25-30% after severe open fractures [14]. MSI not only account for a high morbidity and mortality rate, they also have a substantial socioeconomic impact [15-17]. As the consequences can be life-changing for the patient due to permanent functional loss or even the need for amputation of the affected limb, patient quality-of-life and functional status decrease significantly [18,19,17]. Starting from 2018, the first patients with severe MSI were treated successfully in UZ Leuven with a combination of bacteriophage therapy and antibiotic therapy. The first four cases were recently published by our group [10]. To this day, we have treated eight patients with overall a successful outcome. These patients all had severe infections with no alternative treatment options available (i.e., last resort cases). In UZ Leuven, this has led to a significant increase in the demand and referral of patients with DTT infections.

Chronic rhinosinusitis (CRS):
CRS is an inflammatory disorder of the paranasal sinuses and linings of the nasal cavity, which affects 5-15% of the global population with a significant economic and antibiotic burden, as well as a profound impact on quality-of-life [20]. In addition to antibiotic resistance, the formation of bacterial biofilms in CRS has been associated with more frequent outpatient visits, increased use of antibiotics, disease recurrence following sinus surgery and therapy-refractory CRS [21,22]. Furthermore, the prevalence of CRS in patients with cystic fibrosis (CF) even borders on 100% [23]. In these patients, CRS is also associated with recurrent infections of the lower respiratory tract, which in itself is a risk factor for graft failure after lung transplantation [24,25]. Both *in vitro* studies of clinical isolates and *in vivo* animal models of sinusitis have shown effectiveness of phage cocktails in reducing biofilm formation and treating infections. Similar results were obtained with phages directed against *P. aeruginosa* in patients with CF. Given the association between paranasal sinus colonisation with pathogenic bacteria and recurrent lower respiratory tract infections, phage therapy may help prevent the development of chronic lung infection in these patients [26,27].

Sepsis in the critically ill:
Sepsis is diagnosed in approximately 30% of patients admitted to the intensive care unit (ICU) and is associated with a mortality rate of 26% [28]. Due to the widespread diffusion of MDR pathogens, such as methicillin-resistant *Staphylococcus aureus*, vancomycin-resistant *Enterococcus spp.* and carbapenem resistant Gram-negative bacteria, the most commonly employed antibiotic regimens are often inappropriate, which attributes to an increased morbidity and mortality in these patients [28]. With this in mind, several *in vitro* and *in vivo* studies [29-31], and some first case reports [32,33], have been published on the use of phage therapy for patients diagnosed with sepsis. Recently, the first cases were treated successfully with intravenous phage cocktails and no adverse effects were reported [34].

3. Trial objectives and Design

3.1 Trial objectives

<u>Objective (O) 1.</u> To integrate and optimize phage therapy in three medical disciplines in a continuous and sustainable way, allowing long-term follow-up of patients.

Several case reports and studies on different infectious diseases treated with phage therapy have been published in the past ten years [32,35,36,10,37-40,26]. However, as different approaches and treatment protocols were used, it becomes extremely difficult to compare results and extrapolate treatment protocols to the current standard-of-care. To date, solid evidence on which treatment protocol to use (e.g. phage titer, duration of treatment, systemic (side) effects, etc.) is lacking. Although results of these reports are essentially promising and phage therapy was recently implemented in the legal framework for magistral preparations in Belgium [9], phage therapy is still overall considered as an experimental therapy that can only be used in very specific cases [41]. In UZ Leuven, a **standardized treatment approach** guided by an MPTF (later in this protocol referred to as CBL) was set up for last resort MSI patients [10]. The aim of this project is to expand this approach to CRS and sepsis patients and to set up a **prospective patient registry**. Data will be collected prior to, during and after phage therapy. Patients will be followed up for at least one year. Furthermore, in order to evaluate the added value of phage therapy relative to the standard-of-care, patients for whom no phages could be selected, but who were found eligible by the CBL for phage therapy, will also be included in the registry as a control group.

The regular analysis of this database will provide insight in the **safety and efficacy** of the applied phage therapy protocol and will allow adaptations in the treatment protocols to be made and evaluated (Plan-Do-Check-Act).

O2. *To understand the physiology of phage therapy using three different routes of administration.*

Another reason for the lack of consensus on the optimal treatment protocol for phage therapy is the lack of knowledge regarding the pharmacology behind phage therapy. Pharmacological awareness will not only help to optimize treatment protocols, it will also help to understand the etiology behind treatment failures [42]. For example, via the combined measurement of bacterial burden, bacterial resistance to phages and immunological biomarkers such as antiphage antibodies, we will be able to **describe factors that predict the success of phage treatment**, which can consequently be used to **improve, adapt and personalize future phage therapy.** The aim of O2 is to gain insight into the pharmacology of phage therapy in three different patient populations (and three different routes of administration).

This study aims to answer following questions regarding phage dynamics:

1. What is the safety and tolerability of the phage therapy protocol for each patient population?

To evaluate the safety and tolerability of the applied phage therapy protocol, patients are monitored closely. Their clinical parameters will be collected as detailed in Table 1. Standard blood tests (i.e., complete blood count, C-reactive protein, liver and kidney function tests) are performed that will be collected in the registry as described in Table 1. Even though no phage-related toxicity is expected, by monitoring these parameters closely, in case of abnormalities, quick action can be undertaken. Furthermore, as these parameters are collected in a highly standardized way, it will be possible to compare them between patient populations. This will help us gain insight in the systemic effects of the applied route of administration.

2. What is the efficacy of phage therapy in these patient populations?

To assess the potential of phage therapy to eradicate the infection in combination with standard concomitant therapy, a combination of quantitative and qualitative outcome measures will be evaluated. The most important quantitative measure is the bacterial load during and after phage therapy. This will be assessed in all patients, except for MSI patients where it is not feasible to have multiple deep tissue cultures taken after the wound has been closed. As an alternative, during phage therapy, the draining fluid will be cultured. As the draining tubes are typically removed after phage therapy is stopped, cultures can no longer be obtained during the follow-up period, unless in case of recurrence. The main outcome measure for these patients is a disease-free period of at least one year after treatment. The samples from CRS (nasal swabs and sputum samples) and sepsis (hemocultures) patients are therefore pivotal to understand the effects of phages on bacteria in the follow-up setting. The results from these cultures teach us how long it takes for the phages to eradicate the bacteria. For CRS patients, other specific quantitative outcome measures are available and frequently used in daily practice. These include smell tests, radiological scores (Lund-Mackay CT scoring system) and endoscopic scores (Lund-Kennedy score,Modified Davos score), which will be collected and compared before, during and after treatment. Regarding qualitative outcome measures, patient-reported outcomes (PROs) are collected from CRS and MSI patients. These include the PROMIS (patient-reported outcomes measurement information system) on global health and pain interference (for both CRS and MSI patients), PROMIS on physical

function (for MSI patients) and the Sino-Nasal Outcome Test-22 (SNOT-22) and VAS questionnaires for CRS patients.

3. If still isolated during or after phage therapy, do bacterial resistance mechanisms arise and if so, do the bacteria lose virulence factors?

Bacteria and phages have co-evolved for billions of years. This co-evolution seems to be an important driver of ecological and evolutionary processes in microbial communities (48). The frequency at which phage resistance hinders phage therapy is not clear, although one group reported rates varying from 17% (to Staphylococcal phages) to 85% (to *E. coli* phages) (49). On the other hand, it has been suggested that bacteria developing phage resistance have a high fitness/virulence cost (50), making them more susceptible to other antimicrobial treatments.

Therefore, bacteria that are isolated during and after phage therapy will help us gain insight into resistance development of bacteria against phages, which is especially interesting in case of treatment failures. To understand the impact of phage resistance, the isolated bacteria will be tested for sensitivity against the applied phages and the efficiency of plating will be compared to the initial (pre-treatment) one. Bacterial isolates will also be sequenced to look for genetic mutations causing the increased tolerance to the selected phages and the antibiogram will be compared to the initial one (i.e., to determine if the bacteria have become more susceptible to certain antibiotics). Based on these results, phage cocktails can be adapted.

Following questions regarding phage kinetics will be answered:

1. What is the systemic exposure of phages when applied locally (i.e. in case of MSI) or intranasally (i.e. in case of CRS)?

As it is likely that the systemic exposure of a virulent phage is significantly modified due to its adsorption (i.e. attachment) to the susceptible bacterial cells causing the infection, it becomes difficult to extrapolate the systemic exposure of other antimicrobial drugs to that of phages. Furthermore, virulent phages multiply in their host, and, as a result, while classic antibiotics decay over time, bacteriophage titers increase in the presence of a susceptible host (43). To evaluate the local phage titer, the draining fluid of the MSI patients, and nasal swabs and sputum samples from CRS patients, are collected. After blood drawing (time points, see table 1), samples will be centrifuged and the serum will be collected. Half of the serum will be processed to look for phage presence (phage titration). In all blood samples, phage quantification will be performed using the double agar overlay method (viable fraction) and qPCR (total phage titer in the blood). If viable phages are present, they will be isolated and sequenced to look for mutations. The other half of the serum sample will be used to determine the presence of antiphage antibodies, as discussed below. Results from these assays will be stored in the patient registry.

2. What is the phage kinetic profile of the respective phages when applied systemically (i.e., in case of sepsis) and how long do they remain active?

As described in Table 1, multiple blood (serum) samples are taken daily from the sepsis patients. Phage titration will be performed using the double agar overlay method (viable fraction) and qPCR (total phage titer in the blood). From these samples we can elucidate how long phages stay present in the blood before the next intravenous administration. This information is crucial to evaluate the administration protocol (i.e. whether more or less administrations are required). Also, after phage therapy is stopped,

we can evaluate how long phages persist, in the presence or absence of their bacterial host (i.e., based on the culture results). The bacterial load in the blood will be quantified by determining and extrapolating 'time-to-positivity' of the hemocultures. An important asset of the double agar overlay method is that we can isolate the phage plaques used for phage quantification and sequence them to determine precisely if and when phage mutants arise during intravenous phage therapy. The latter will be further explained below.

3. Does phage therapy induce neutralizing antibodies?

The clearance of phages by the immune system may affect the efficacy of phage therapy (42, 44). Since phages are encountered on a daily basis (e.g. through various foods), low titers of phage-specific antibodies are common in patients, but titers may increase during phage therapy. The induction of the innate immune system, that clears phages through phagocytosis (i.e. the reticuloendothelial system), as well as of the adaptive immune system by the production of phage-neutralizing antibodies, has been associated with early depletion of phages and subsequent impairment of efficacy (45, 46). For oral and topical applications, this seems to be less of an issue compared to systemic application. However, it may still be necessary to compensate for this phenomenon by repeating phage administration, increasing phage titer or using different phages or a phage cocktail (44-47). Further exploration of this topic is needed to elucidate the role of the human immune response in phage therapy. Therefore, from all three patient populations, serum samples will be processed using the phage neutralization assay as previously described (10) to detect antiphage antibodies on the time points listed in Table 1.

O3. *To characterize the interaction between phage and bacteria and optimize future phage cocktails by applying a genome-based approach.*

The high level of specificity of phages, targeting only a fraction of strains within a bacterial species, has two main consequences:

1) Host susceptibility is a key exclusion factor that requires expansion of phage banks.

2) Combining phages into a single cocktail is needed.

However, there are no principle-based guidelines to ascertain the synergy/antagonism between phages. Blood samples and cultures are generally and frequently taken during and after phage therapy. In this project, phages and bacteria that are isolated from these samples will be analysed on a genomic level. The valuable information regarding the interaction between phage and bacteria that is thus obtained will be used to **expand and evolve available phages** and **optimize a machine-learning strategy** based on genomics data of the phage/bacteria to **enhance personalized cocktail composition**.

3.2 Primary endpoints

To gain insight in the safety and tolerability of phage therapy for each route of administration

3.3 Secondary endpoints

To gain insight in the efficacy of phage therapy for each indication, using standardized treatment protocols.

3.4 Trial Design

This is a prospective, observational, academic study.

3.5 Study diagram

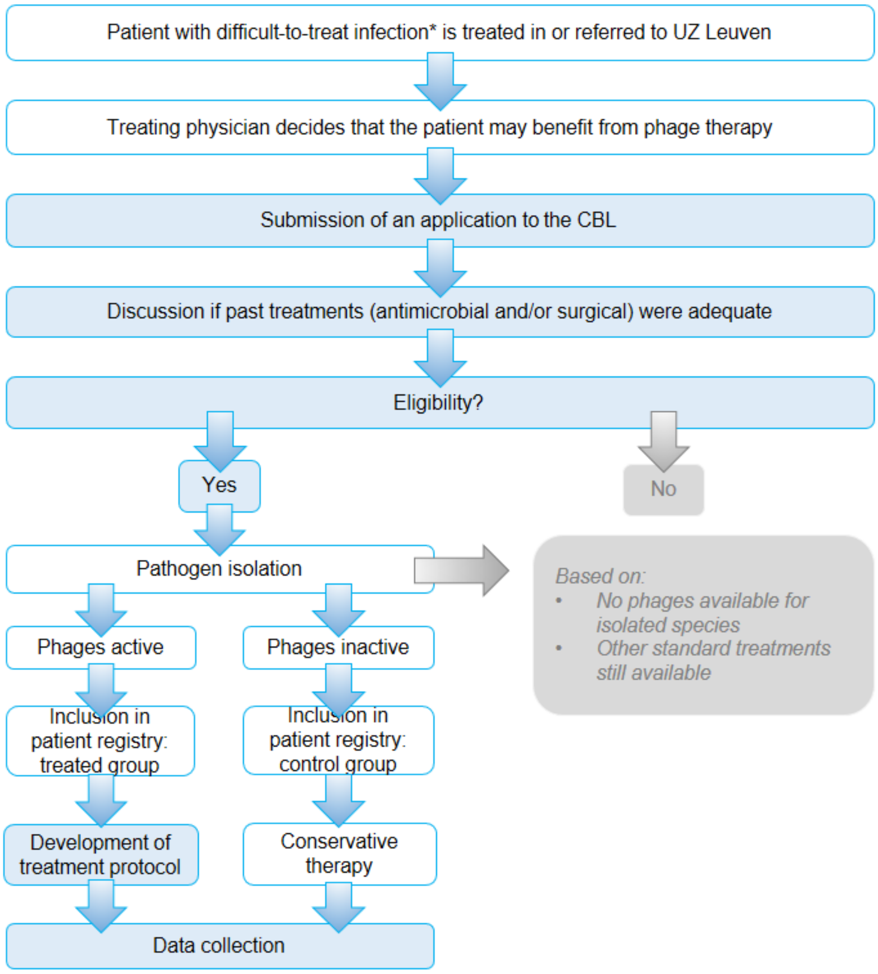

Figure 1. Application procedure for bacteriophage therapy in UZ Leuven. To qualify for phage therapy, the medical history and prior/current treatment regimens have to be evaluated by the CBL for each individual patient.

Study flowchart

Application procedure

When the treating physician believes his/her patient may benefit from phage therapy, it is standard practice to present and discuss the patient with the CBL (Figure 1). The CBL consists of following members (the names that are provided here represent the main responsible persons for each discipline, other members may be added throughout the study):

- ID physicians/intensive care specialists (Willy Peetermans, Paul De Munter, Yves Debaveye)
- Pneumologist (Lieven Dupont)
- Clinical pharmacists (Isabel Spriet)
- Microbiologists (Melissa Depypere)
- Surgeons (Willem-Jan Metsemakers, Laura Van Gerven)
- Phage scientists (Rob Lavigne, Jean-Paul Pirnay, Maya Merabishvili)

Eligibility screening is performed by the CBL. This group will look into the medical history of each patient, and will determine if past treatments were adequate and if there are any other (surgical or antibiotic) treatment options available. A report of this discussion will be documented in the patient's medical file as a 'multidisciplinair contact'. If there are no alternative (surgical or antibiotic) treatment options available (i.e., last resort cases), the patient is eligible for inclusion in the PHAGEFORCE study.

Informed consent will be asked from the patient, either at the outpatient clinic or at the hospital, if the patient is hospitalized. After informed consent, bacteriological cultures (if no recent cultures available, (intraoperative) cultures will be taken from MSI and CRS patients and hemocultures will be taken from sepsis patients) will be sent to the MHQA for a phagogram. Depending on the susceptibility of the isolates the CBL will decide if the patient can be included in the phage treated group or the control group. The CBL will furthermore set up the treatment plan accordingly and will document this in the patient's medical file (referred to as CBL's final treatment plan). Therefore, in this study, there are two types of standard-of-care, as displayed in Figure 2 and defined below:

When the patient is included in the phage treatment group (i.e., the isolated pathogens are susceptible to the available phages), the CBL will design the treatment protocol and document it in the CBL's final treatment plan, which is also documented in the patient's medical file.

The phages that will be used, are produced according to the monograph (attached to this protocol) at the Queen Astrid Military Hospital (QAMH). Furthermore, these phages are well-characterized, in that the genetic sequence and therefore the confirmation of each phage's strictly lytic profile and absence of undesired genetic determinants such as toxin and antibiotic resistance genes (i.e., genetic passport) are available. The analyses to obtain such passports are performed by Sciensano, the federal research institute for public health. Also, each batch that is produced at the QAMH is analyzed by Sciensano before it can be used in humans.

Before and during phage treatment, it is standard to have several blood tests and samples analyzed (as listed in Table 1) to monitor the general health status of the patient. Follow-up via the outpatient clinic will also be according to standard of care for phage treated patients until 3 months after final phage administration and thereafter according to the standard of care for the underlying pathology. The CBL will also set up the treatment protocol for Control patients (standard treatment for the end-stage infection). Control patients will only be subjected to blood analyses and sampling when required according to the standard of care for the underlying pathology. They will be followed for one year from

the CBL's final treatment plan. The results of all blood tests and culture tests that are performed in that period with regards to the underlying infection, will be recorded in the patient registry. All eligible MSI and CRS patients will also be asked to fill out PROMIS (for MSI and CRS) or SNOT-22 and VAS (CRS) questionnaires at standard follow-up outpatient visits.

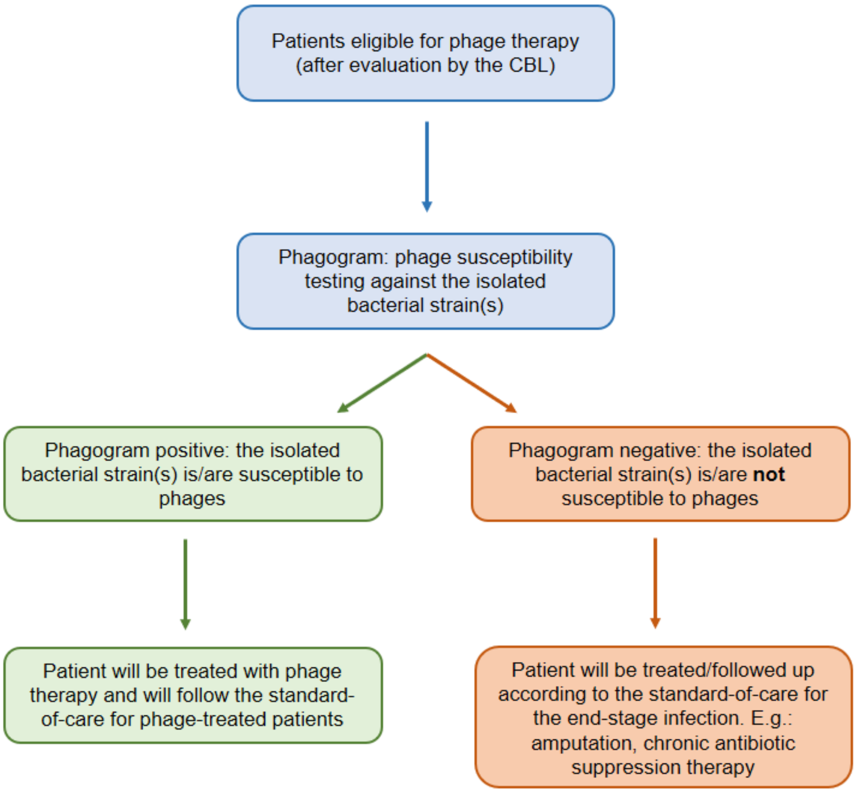

Figure 2. Depending on the result of the phagogram, the patient will follow either the phage therapy trajectory (green) or the control trajectory (orange). For each trajectory, there is a standard-of-care.

Data collection	MSI		CRS		Sepsis	
	**Positive phagogram**	**Negative phagogram**	**Positive phagogram**	**Negative phagogram**	**Positive phagogram**	**Negative phagogram**
**ICF**	After positive evaluation CBL, in the outpatient clinic or at the hospital ward (if the patient is already hospitalized)					
**Physical exam**	Prior to and after each phage administration<br><br>Two-weekly, until 3 months after final administration<br><br>According to standard-of-care for the MSI thereafter	According to standard-of-care for the MSI	Physical exam including nasal endoscopy. At day of inclusion and during every follow-up consultation (day 1, day 5-7 during phage therapy, 1 week, 1 month, 3 months after final phage administration). Physical exam will be performed prior to and 30 min after the first phage administration.<br><br>According to standard-of-care for CRS thereafter	According to standard-of-care for CRS	Prior to and after each phage administration<br><br>Weekly, until 4 weeks after final administration<br><br>Thereafter, two-weekly until 3 months after final administration<br><br>According to standard of care for sepsis thereafter	According to standard-of-care for sepsis
**Blood analysis**	Before start of phage therapy (max 2 days before first administration)<br><br>During phage therapy: Day 1, Day 2, Day 4, Day 7, Day 10<br><br>Two-weekly, until 3 months after final administration<br><br>According to standard-of-care for the MSI thereafter	According to standard-of-care for the MSI	At day of inclusion and during the follow-up consultation (after 5-7 days of phage therapy, 1 week, 1 month and 3 months after final phage administration).<br><br>According to standard-of-care for CRS thereafter	According to standard-of-care for CRS	Before start of phage therapy (max 2 days before first administration)<br><br>Daily during phage therapy<br><br>Weekly, until 4 weeks after final administration<br><br>Thereafter, two-weekly until 3 months after final phage administration<br><br>According to standard of care for sepsis thereafter	According to standard of care for the underlying disease
**Serum collection***	Before start of phage therapy (max 2 days before first administration)<br><br>During phage therapy: Day 1, Day 2, Day 4, Day 7, Day 10<br><br>Two-weekly, until 3 months after final administration	N/A	At day of inclusion and during the follow-up consultation (after 5-7 days of phage therapy, 1 week, 1 month and 3 months after final phage administration).	N/A	Before start of phage therapy (max 2 days before first administration)<br><br>Daily during phage therapy (before and after administration (0, 15, 30 min), after 1h, 1.5h, 4h, 6h)<br><br>Every 6 hours in the week following the final phage administration<br><br>Weekly, until 4 weeks after final administration<br><br>Thereafter, two-weekly until 3 months after final phage administration	N/A

**Parameters**	Prior to and after each phage administration<br><br>Two-weekly, until 3 months after final phage administration<br><br>According to standard-of-care for the MSI thereafter	According to standard-of-care for the MSI	Prior to the first phage application and 30 minutes afterwards.<br>During every follow-up consultation (day 5-7 of phage therapy, 1 week, 1 month and 3 months after final phage administration).<br><br>According to standard-of-care for CRS thereafter	According to standard-of-care for CRS	Prior to and after each phage administration<br><br>Weekly, until 4 weeks after final administration<br><br>Thereafter, two-weekly until 3 months after final phage administration<br><br>According to standard of care for sepsis thereafter	According to standard of care for sepsis
**Other samples****<br>Deep tissue cultures (MSI)<br>Draining fluid (MSI)<br>Nasal swabs/sputum samples (CRS)<br>Hemocultures (sepsis)	Deep tissue cultures: upon indication<br><br>Draining fluid: after each phage administration	Deep tissue cultures: upon indication	Nasal swab to detect bacteria: Before the first application of phages. Afterwards during every follow-up consultation (day 5-7 of phage therapy, 1 week, 1 month and 3 months after final phage administration).<br><br>Nasal swab to detect phages: day 5-7 of phage therapy (preferably before the first administration of the day), and 1 week after final phage administration<br><br>Sputum sample (only in patients with cystic fibrosis): at day of inclusion, 1 month after final phage administration<br><br>According to standard-of-care for CRS thereafter	Nasal swabs/sputum samples: upon indication	Hemocultures: the day before the start of phage therapy, prior to and after the first infusion, daily for the entire duration of phage therapy<br><br>Weekly, (or more frequently upon indication) until 4 weeks after final phage administration;<br><br>Thereafter two-weekly until 3 months after final administration.<br><br>Upon indication thereafter	Hemocultures: upon indication
**Patient questionnaires**<br>PROMIS global health, pain interference (MSI, CRS), physical function (MSI)<br>SNOT-22 and VAS score (only CRS)	At the outpatient clinic or hospital ward, after signing of ICF (after positive evaluation CBL)<br><br>At the end of treatment (day of final phage administration)<br><br>At follow-up consultation: 6 weeks, 12 weeks, 6 months and 12 months after final phage administration.	At the outpatient clinic or hospital ward, after signing ICF (after positive evaluation CBL)<br><br>At follow-up consultation: 6 weeks, 12 weeks, 6 months and 12 months after CBL's final treatment plan	At the outpatient clinic, after signing of ICF (after positive evaluation CBL)<br><br>1 week, 1 month and 3 months after final phage administration	At the outpatient clinic or hospital ward, after signing of ICF (after positive evaluation CBL), 1 week, 1 month and three months after CBL's final treatment plan	N/A	N/A
**Scoring systems (standard performed**	Radiological evaluation: at the outpatient clinic or hospital ward, after	Radiological evaluation:	Baseline nasal endoscopy, using Lund-Kennedy and Modified Davos	Baseline nasal endoscopy, using	SOFA-score:	SOFA-score:

**and documented by the treating physician/radiologist)**<br>Radiological evaluation (MSI)<br>Scoring systems for nasal endoscopy and imaging (CRS)<br>SOFA-score (sepsis)	signing of ICF (after initial positive evaluation CBL), before start of phage therapy<br><br>At the end of treatment (day of final phage administration)<br><br>According to the standard of care for the MSI thereafter	At the outpatient clinic or hospital ward, after signing ICF (after initial positive evaluation CBL),<br><br>according the standard of care for the MSI thereafter	scoring system, within 3 months prior to signing of ICF. Afterwards during every follow-up consultation (day 1, day 5-7 of phage therapy; 1 week, 1 month and three months after final phage administration).<br><br>CT sinuses with Lund-Mackay score: CT-scan should be carried out at least <3 months before start of phage therapy (or after recent surgery). Afterwards CT-scan should be repeated according to the standard-of-care for CRS<br><br>EPOS criteria for defining controlled and uncontrolled CRS: before start of treatment, at 1 month and at 3 months after first application of phages	Lund-Kennedy scoring system within 3 months prior to signing of ICF.<br><br>Lund-Mackay score:<br>At the outpatient clinic or hospital ward, after signing of ICF (after positive evaluation CBL)<br><br>According to standard-of-care for CRS	At the hospital ward, after signing of ICF (after positive evaluation CBL)<br><br>Daily until discharge	At the hospital ward, after signing of ICF (after positive evaluation CBL)<br><br>Daily until discharge
**Other (CRS)**			Allergy testing: should be carried out before start of phage therapy (recent testing <5 years ago)<br><br>Smell-testing: Sniffin' sticks at day of inclusion and 3 months after start of phage therapy			

Table 1. Data to be collected according to type of infection and result of the phagogram.

*: These samples will be used to determine if any neutralizing antibodies against the applied phages are induced, which may interfere with the phage therapy outcome. These samples will also be used to perform phage titration.

**: These samples will be cultured and analysed for phage presence/phage titration. In case of the cultures, all isolated pathogens will be stored at -80°C for further analysis (sequencing). From draining fluid samples, a phage titration assay will be performed.

4. Selection and withdrawal of subjects

4.1 Inclusion criteria

All patients:

- Diagnosed with an MSI or CRS or sepsis, and
- For whom all previous treatments (surgical and antibiotic) have failed or for whom no other treatment options are available (i.e., last resort cases, based on the assessment of the CBL), for example in case of bacterial resistance. And
- Of whom the pathogen causative for the infection is one for which phages are available in the phage bank, and
- Who have given informed consent to have their data collected in a patient registry

4.2 Exclusion criteria

All patients:

- With an infectious disease other than MSI, CRS or sepsis. And, or
- For whom standard treatment alternatives are still available. And, or
- Of whom the pathogen causative for the infection is not one for which phages are available in the phage bank. And, or
- Who refused to give their informed consent

4.3 Expected duration of trial

Patients will be included in the study for a total of four years. Since each patient will be followed up for a year after the final administration of phages or after the CBL's final treatment plan (for patients for whom no phages can be selected (control patients)), the total duration of the study will be maximum 5 years.

5. Trial Procedures

All trial procedures are according to the standard of care (either for phage therapy or for the underlying infection) (Figure 2, Table 1).

5.1 By visit

5.1.1. Musculoskeletal infections (MSI)

ICF: Depending on the clinical status of the patient, this may be at the hospital ward (when the patient is hospitalized) or at the outpatient clinic.

For patients who are treated with phage therapy

Upon admission/max. 2 days prior to the start of phage therapy (baseline):

- Physical exam
- Blood tests
- Serum collection
- Parameter collection
- Questionnaires
- Radiological evaluation

Daily during phage therapy:

- Physical exam: before and after each phage administration
- Parameter collection: before and after each phage administration
- Draining fluid collection

During phage therapy: day 1, 2, 4, 7, 10:

- Blood tests
- Serum collection
- Parameter collection
- Monitoring of adverse events

End of phage therapy:

- Questionnaires
- Radiological evaluation

Two-weekly after final phage administration (at the outpatient clinic or in case the patient is still hospitalized, at the hospital ward) until three months after the final phage administration:

- Physical exam
- Blood tests
- Serum collection
- Parameter collection
- Radiological evaluation upon indication
- Monitoring of adverse events

<u>In patients who are eligible for phage therapy, but for whom no phages are available (based on the phagogram) and starting from 3 months after the final phage administration for patients who could be treated with phage therapy:</u>

- Physical exams, blood tests, serum and parameter collection will be performed according to the standard of care for the MSI (at the outpatient clinic, or when (still) hospitalized, at the hospital ward)
- Deep tissue cultures will be taken upon indication
- Questionnaires and scoring systems will be collected at 6 weeks, 12 weeks, 6 months and 12 months after the final decision of the CBL. For patients who were treated with phage therapy, questionnaires and scoring systems will be collected at 3 months, 6 months and 12 months after the final phage administration.

5.1.2. Sepsis

ICF: Signing of ICF at the hospital ward

For patients who are treated with phage therapy

Upon admission/max 2 days prior to the start of phage therapy (baseline):

- Physical exam
- Blood tests
- Serum collection
- Hemocultures: the day before the start of phage therapy
- Parameter collection
- Scoring system: SOFA

Daily during phage therapy:

- Physical exam: before and after each phage administration
- Blood tests
- Serum collection: before and after each phage administration
- Parameter collection: before and after each phage administration
- Hemocultures: before and after the first phage administration and daily during phage therapy
- Scoring system: SOFA
- Monitoring of adverse events

Weekly after final phage administration (at the outpatient clinic or in case the patient is still hospitalized, at the hospital ward) until four weeks after the final phage administration and two-weekly thereafter until 3 months after the final phage administration:

- Physical exam
- Blood tests
- Serum collection
- Parameter collection
- Hemocultures
- Scoring system: SOFA
- Monitoring of adverse events

In patients who are eligible for phage therapy, but for whom no phages are available (based on the phagogram) and starting from 3 months after the final phage administration for patients who could be treated with phage therapy:

- Physical exams, blood tests, serum and parameter collection will be performed according to the standard of care for the underlying infection (at the outpatient clinic, or when (still) hospitalized, at the hospital ward)
- Cultures will be taken according to the standard of care for the underlying infection (at the outpatient clinic, or when (still) hospitalized, at the hospital ward)
- Scoring systems will be collected according to the standard of care for the medical discipline and type of infection (at the outpatient clinic, or when (still) hospitalized, at the hospital ward

5.1.3. Chronic rhinosinusitis (CRS)

ICF: The informed consent form will be signed at the outpatient clinic.

For patients who are be treated with phage therapy

Before start of phage therapy (baseline):

- Parameter collection
- Nasal endoscopy: **Lund-Kennedy and Modified Davos** score
- Allergy testing: if performed within 5 years prior to the start of phage therapy, the results of this test will be recorded and no new test will be performed.
- Smell test
- Blood sample
- CT-scan of the sinuses: the most recent CT-scan can be taken, if it was carried out within 3 months prior to the start of phage therapy or after recent surgery. The **Lund-Mackay score** will be evaluated.
- Questionnaire including **SNOT-22-score, VAS-score, PROMIS questionnaires (cfr. MSI)**
- General score (based on medical history, nasal endoscopy and CT-scan): **EPOS criteria for defining controlled and uncontrolled CRS**
- *In case of cystic fibrosis: sputum sample*

Start of phage therapy (day 1):

- Vital parameter collection before start of treatment and 30 minutes after first phage administration
- Physical exam before start of treatment and 30 minutes after first phage administration
- Nasal endoscopy with nasal swab (bacterial) at the middle meatus
- Instructions on how to rinse: first time rinsing together with investigator
- Patient will receive phage vials, rinsing solution and brochure with summary of instructions

Follow-up consultation during phage therapy (day 5-7)

- Parameter collection
- Physical exam
- Nasal endoscopy: **Lund-Kennedy score**
- Nasal swab at middle meatus
- Blood tests
- Serum collection
- Monitoring adverse events

Follow-up consultations (1 week, 1 month, 3 months after final phage administration):

- Vital parameters
- Physical exam
- Nasal endoscopy: **Lund-Kennedy and Modified Davos score**
- Nasal swab at middle meatus
- Blood tests
- Serum collection

- Questionnaires including **SNOT-22-score, VAS-score, PROMIS**
- Monitoring adverse events
- CT-scan: only if indicated by medical doctor (according to the standard-of-care)
- Smell test (only at 3 month outpatient visit)
- *In case of cystic fibrosis: sputum sample (only at 1 week after final phage administration)*

In patients who are eligible for phage therapy, but for whom no phages are available (based on the phagogram) and starting from 3 months after the final phage administration for patients who could be treated with phage therapy:

- Physical exams, blood tests, serum and parameter collection will be performed according to the standard of care for the CRS (at the outpatient clinic)
- Cultures will be taken according to the standard of care for the CRS (at the outpatient clinic)
- Questionnaires and scoring systems will be collected 1 week, 1 month and 3 months after the final decision of the CBL.

5.2 Laboratory tests

Following blood laboratory tests are required for the monitoring (and optimization) of the phage therapy regimen and are therefore considered standard-of-care for patients treated with phage therapy:

- Complete blood count
- Basic metabolic panel
- Inflammatory parameters
- Lactic acid, Creatine kinase
- Liver function tests
- Antiphage-antibodies (serum)
- Phage titration and isolation from draining fluid or blood (these phages will be sequenced)

Several bacterial cultures will be taken from all patients. From MSI patients, the draining fluid will be cultured, nasal swabs and sputum samples will be taken from CRS patients and hemocultures will be obtained from sepsis patients. The isolated pathogens from these cultures will be sequenced (see Section 14 'Translational research').

For patients who are eligible for phage therapy, but for whom no phages are available at the time of testing (based on the phagogram), blood laboratory tests and culturing will be performed and collected according to the standard of care for the underlying infection.

5.3 Other investigations

Radiological examinations

Radiological examinations will be performed according to the standard of care for phage treated patients and for patients who are eligible for phage therapy but for whom no phages are available (Table 2, scoring systems).

6. Assessment of efficacy

- Bacterial load

To assess the potential of phage therapy to eradicate the infection in combination with standard concomitant therapy, a combination of quantitative and qualitative outcome measures will be evaluated. The most important quantitative measure is the bacterial load during and after phage therapy. This will be assessed in all patients, except for MSI patients where it is not feasible to have multiple deep tissue cultures taken after the wound has been closed. As an alternative, during phage therapy, the draining fluid will be cultured. As the draining tubes are typically removed after phage therapy is stopped, cultures can no longer be obtained during the follow-up period, unless in case of recurrence. The main outcome measure for these patients is a disease-free period of at least one year after treatment. The samples from CRS (nasal swabs and sputum samples) and sepsis (hemocultures) patients are therefore pivotal to understand the effects of phages on bacteria in the follow-up setting. The results from these cultures will teach us how long it takes for the phages to eradicate the bacteria.

- Scoring systems

For CRS patients, other specific quantitative outcome measures are available and frequently used in daily practice. These include endoscopic (Modified Davos, Lund-Kennedy), radiological (Lund-Mackay) and combined (EPOS criteria for controlled or uncontrolled CRS) scoring systems, which will be collected and compared before, during and after treatment.

For sepsis patients, the **SOFA score** will be recorded before and after treatment. The SOFA score is an internationally used score to objectively determine the degree of multiple organ failure in critically ill patients. A (rapid) decrease of the SOFA score (delta SOFA) is associated with a better survival and vice versa. Delta SOFA is defined as the difference between the SOFA score at baseline (prior to phage therapy) and the SOFA score at the final day of treatment.

Regarding qualitative outcome measures, **patient-reported outcomes** (PROs) will be collected from CRS and MSI patients. These include the PROMIS (patient-reported outcomes measurement information system) on global health and pain interference (for both CRS and MSI patients), PROMIS on physical function (for MSI patients) and the Sino-Nasal Outcome Test-22 (SNOT-22) and VAS questionnaires for CRS patients).

- Other outcome measures

To evaluate the olfactory function in CRS patients, smell tests (Sniffin' Stick test) will be carried out before and after phage therapy.

7. Assessment of Safety

7.1 Specification, timing and recording of safety parameters

As discussed in the sections above, one of the main aims of this study is to gain insight in the safety profile of the applied phage therapy protocols. Patients will therefore be monitored closely during phage therapy. Their clinical parameters will be collected as detailed in Table 1, and stored in the patient registry. Standard blood tests will be performed frequently and collected in the patient registry. Even though no phage-related toxicity has been described or is to be expected, by monitoring these parameters closely, in case of abnormalities, quick action can be undertaken. In this study, only adverse events/complications directly related to the condition or treatment of the infection are collected. In case an AE or SAE arises during the study period, this will be documented in the eCRF. A data safety monitoring board is set up for this study, which consists of following members:

- ID physicians/intensive care specialists (Eric Van Wijngaerden, Jan Gunst)
- Pneumologist (Pascal Van Bleyenbergh)
- Clinical pharmacists (Peter Declercq)
- Microbiologists (Stefanie Desmet)
- Surgeons (Stefaan Nijs, Mark Jorissen)

For every 25 patients who are included, a report will be provided to the DSMB. They will go over any possible AEs, medication switches, etc. and provide feedback.

7.2 Procedures for recording and reporting adverse events (AE)

7.2.1. Definitions

7.2.1.1. Adverse event (AE)

An AE is any untoward medical occurrence in a patient or subject during an experiment, and which does not necessarily have a causal relationship with this treatment.

An AE can therefore be any unfavourable and unintended sign (including an abnormal laboratory finding), symptom or disease temporally associated with the use of a product, whether or not considered related to the product. Any worsening (i.e., any clinically significant adverse change in the frequency or intensity of a pre-existing condition) should be considered an AE.

In this observational study, AE's related to the infection and treatment of the infection will be recorded during the first three months.

Specifically for sepsis patients:

Patients with sepsis are critically ill patients who are inherently suffering from multiple medical conditions. In daily clinical ICU practice, all medical events and procedures are recorded in the central digital patient file. In this study, only those safety parameters which could theoretically be related to the administration of bacteriophages will be recorded in the patient's digital medical record and in the eCRF. Reporting all untoward medical events on the ICU would not be practically feasible and relevant for the data, given the population heterogeneity and its inherent susceptibility to various medical events and complications.

7.2.1.3. Serious adverse event (SAE)

An SAE is any untoward medical occurrence that results in any of the following:

- Death
- A life-threatening[a] experience
- In-patient hospitalisation or prolongation of existing hospitalisation
- A persistent or significant disability or incapacity
- A congenital anomaly or birth defect
- Important medical events that may be considered an SAE when - based on appropriate medical judgement - they may jeopardise the subject and may require medical or surgical intervention to prevent one of the above outcomes

[a] The term "life threatening" in the definition of SAE refers to an event in which the subject was at risk of death at the time of the event. It does not refer to an event which hypothetically might have caused death if it was more severe.

7.2.1.4. Adverse events of special interest (AESI)

To our knowledge, there are no pre-defined AESIs for phage therapy.

7.2.1.5. Adverse events that do not require reporting

In general, the following should not be reported as AEs:

- Pre-existing conditions, including those found as a result of screening (these should be reported as medical history or concomitant illness).
- Pre-planned procedures unless the condition for which the procedure was planned has worsened from the first trial-related activity after the subject has signed the informed consent.

These events will be recorded in the patient's medical notes according to routine practice.

The following events not to be considered as SAEs are:

- Pre-planned hospitalisations unless the condition for which the hospitalisation was planned has worsened from the first trial-related activity after the subject has signed the informed consent.
- Hospitalisation as part of a standard procedure for protocol therapy administration. However, hospitalisation or prolonged hospitalisation for a complication of therapy administration will be reported as an SAE.
- Hospitalisation or prolongation of hospitalisation for technical, practical, or social reasons, in absence of an AE.

7.2.2. Recording and reporting of AEs

The investigators and study personnel will seek information on AEs during each patient contact until three months after final phage administration or after the CBL's final treatment plan. All events that can be related to the infection or treatment of infection, whether reported by the patient or noted by study personnel, will be recorded in the patient's medical record and in the eCRF within a reasonable time after becoming aware. If available, the diagnosis will be reported on the AE page, rather than the individual signs or symptoms. If no diagnosis is available, the investigator records each sign and symptom as individual AEs.

Following information will be recorded for each AE:

- AE description
- start and stop date of the AE
- detailed information about the additional treatment(s) performed due to the occurrence of the AE
- severity
- seriousness
- outcome

An overview of treated patients and their possible side effects will be provided and discussed by the DSMB.

7.2.2.1. Assessment

All AEs will be evaluated by an Investigator as to:

- **Seriousness:** whether the AE is an SAE. See above for the seriousness criteria.
- **Severity:**
  - Severity must be evaluated by an Investigator according to the following definitions:
    - *Mild* – no or transient symptoms, no interference with the subject's daily activities
    - *Moderate* – marked symptoms, moderate interference with the subject's daily activities
    - *Severe* – considerable interference with the subject's daily activities, unacceptable

7.2.3. Follow-up of adverse events

Each AE will be followed up until resolved with or without persistent damage or until the end of the patient's study participation, whichever occurs first.

8. Statistics

8.1 Sample size

Since data on the application of phages for the above-mentioned indications is currently scarce, we are unable to perform a power analysis due to the absence of an objective effect size. The main aim of this study is to obtain extensive safety data and describe preliminary efficacy data. Based on the applications for phage therapy we currently receive, we estimate to include a total of approximately 150 patients of which approximately 75 will have positive phagograms and can thus be treated with phage therapy.

8.2 Analysis

As the aim of this registry is mainly descriptive and exploratory, patient characteristics and outcomes recorded at standard of care scheduled follow-up assessments will be presented using simple summary statistics.

Categorical variables will be summarized using the frequency and percentage for each category. Continuous variables will be summarized using the mean, standard deviation, inter-quartile range, and minimum and maximum values. These summary statistics will in addition be presented according to clinically relevant categories, i.e. according to treatment received.

Complications will be reported both at the patient level and AE level.

Given the exploratory nature of this study, any results will have to be interpreted carefully.

9. Direct access to source data and documents

The investigators permit trial-related monitoring, audits, EC review, and regulatory inspections (where appropriate) by providing direct access to source data and other documents (ie patients' case sheets, blood test reports, X-ray reports, etc).

10. Ethics and regulatory approvals

The study will be conducted in compliance with the principles of the Declaration of Helsinki (2013), the principles of GCP and will be in accordance with all applicable regulatory requirements. This protocol and related documents will be submitted for review to the Medical Ethics Committee of the University Hospitals Leuven (Ethische Commissie Onderzoek UZ/KU Leuven).

The Investigators and University Hospitals Leuven shall treat all information and data relating to the study disclosed to the University Hospitals Leuven and/or Investigator in this study as confidential and shall not disclose such information to any third parties or use such information for any purpose other than the performance of the Study. The collection, processing and disclosure of personal data, such as patient health and medical information is subject to compliance with applicable personal data protection and the processing of personal data (General Data Protection Regulation (EU 2016/679)).

11. Data Handling

Data will be submitted to an eCRF. Patient data are coded, implying there continues to be a link between the data and the individual who provided it. The research team is obligated to protect the data from disclosure outside the research according to the terms of the research protocol and the informed consent document. The subject's name or other identifiers will be stored separately from their research data and replaced with a unique code to create a new identity for the subject.

12. Data Management

For this registry-based study, an eCRF will be designed using RedCap to accommodate all study-specific features. Access to the eCRF is password protected and specific functions are assigned (e.g. Study coordinator, investigator, CRA, etc.). The eCRF is completed in a timely manner after a patient's visit. Subjects' identities will be coded by giving each patient a unique patient identifier.

13. Translational research

Phage titration is performed at the Laboratory of Gene Technology (LoGT), under supervision of prof. Rob Lavigne. Draining fluid and serum from phage treated patients will be analyzed. Phage titration will be performed in these samples. Draining fluid samples from MSI patients will also be cultured. The phages and bacteria that are isolated from these samples, will be sequenced.

14. Publication Policy

Results will be published with careful attention to maintain study subject anonymity. Any participating discipline will be able to use their own data for non-commercial internal and educational purposes (e.g. annual summary reporting, presentations at symposia or meeting) and publication of such data in theses or dissertations. However, if a participating discipline wants to use its own data to publish in a peer-reviewed journal, national and international conferences publishing abstracts in journals, it will require approval by all involved investigators.

15. Insurance/Indemnity

In accordance with the Belgian Law relating to experiments on human persons dated May 7, 2004, Sponsor shall assume, even without fault, the responsibility of any damages incurred by a Study Patient and linked directly or indirectly to the participation to the Study, and shall provide compensation therefore through its insurance."

16. Financial Aspects

Translational research: Interdisciplinary Networks grant (KU Leuven)

17. References

1. WHO (2015) Global action plan on antimicrobial resistance World Health Organization
2. Onsea J, Wagemans J, Pirnay JP, Di Luca M, Gonzalez-Moreno M, Lavigne R, Trampuz A, Moriarty TF, Metsemakers WJ (2020) Bacteriophage therapy as a treatment strategy for orthopaedic device-related infections: where do we stand? . European cells & materials 39:193-210. doi:10.22203/eCM.v039a13
3. Kutter E, De Vos D, Gvasalia G, Alavidze Z, Gogokhia L, Kuhl S, Abedon ST (2010) Phage therapy in clinical practice: treatment of human infections. Current pharmaceutical biotechnology 11 (1):69-86
4. Pires DP, Oliveira H, Melo LD, Sillankorva S, Azeredo J (2016) Bacteriophage-encoded depolymerases: their diversity and biotechnological applications. Applied microbiology and biotechnology 100 (5):2141-2151. doi:10.1007/s00253-015-7247-0
5. Abedon ST, Kuhl SJ, Blasdel BG, Kutter EM (2011) Phage treatment of human infections. Bacteriophage 1 (2):66-85. doi:10.4161/bact.1.2.15845
6. Abedon ST, Thomas-Abedon C (2010) Phage therapy pharmacology. Current pharmaceutical biotechnology 11 (1):28-47
7. Loc-Carrillo C, Abedon ST (2011) Pros and cons of phage therapy. Bacteriophage 1 (2):111-114. doi:10.4161/bact.1.2.14590
8. Pirnay JP, De Vos D, Verbeken G, Merabishvili M, Chanishvili N, Vaneechoutte M, Zizi M, Laire G, Lavigne R, Huys I, Van den Mooter G, Buckling A, Debarbieux L, Pouillot F, Azeredo J, Kutter E, Dublanchet A, Gorski A, Adamia R (2011) The phage therapy paradigm: pret-a-porter or sur-mesure? Pharmaceutical research 28 (4):934-937. doi:10.1007/s11095-010-0313-5
9. Pirnay JP, Verbeken G, Ceyssens PJ, Huys I, De Vos D, Ameloot C, Fauconnier A (2018) The Magistral Phage. Viruses 10 (2). doi:10.3390/v10020064
10. Onsea J, Soentjens P, Djebara S, Merabishvili M, Depypere M, Spriet I, De Munter P, Debaveye Y, Nijs S, Vanderschot P, Wagemans J, Pirnay JP, Lavigne R, Metsemakers WJ (2019) Bacteriophage Application for Difficult-to-treat Musculoskeletal Infections: Development of a Standardized Multidisciplinary Treatment Protocol. Viruses 11 (10). doi:10.3390/v11100891
11. Cram P, Lu X, Kates SL, Singh JA, Li Y, Wolf BR (2012) Total knee arthroplasty volume, utilization, and outcomes among Medicare beneficiaries, 1991-2010. Jama 308 (12):1227-1236. doi:10.1001/2012.jama.11153
12. Kurtz SM, Lau E, Watson H, Schmier JK, Parvizi J (2012) Economic burden of periprosthetic joint infection in the United States. The Journal of arthroplasty 27 (8 Suppl):61-65.e61. doi:10.1016/j.arth.2012.02.022
13. Patel AA, Buller LT, Fleming ME, Chen DL, Owens PW, Askari M (2015) National trends in ambulatory surgery for upper extremity fractures: a 10-year analysis of the US National Survey of Ambulatory Surgery. Hand (N Y) 10 (2):254-259. doi:10.1007/s11552-014-9703-1
14. Papakostidis C, Kanakaris NK, Pretel J, Faour O, Morell DJ, Giannoudis PV (2011) Prevalence of complications of open tibial shaft fractures stratified as per the Gustilo-Anderson classification. Injury 42 (12):1408-1415. doi:10.1016/j.injury.2011.10.015

15. Hak DJ, Fitzpatrick D, Bishop JA, Marsh JL, Tilp S, Schnettler R, Simpson H, Alt V (2014) Delayed union and nonunions: epidemiology, clinical issues, and financial aspects. Injury 45 Suppl 2:S3-7. doi:10.1016/j.injury.2014.04.002
16. Olesen UK, Pedersen NJ, Eckardt H, Lykke-Meyer L, Bonde CT, Singh UM, McNally M (2017) The cost of infection in severe open tibial fractures treated with a free flap. International orthopaedics 41 (5):1049-1055. doi:10.1007/s00264-016-3337-6
17. Metsemakers WJ, Onsea J, Neutjens E, Steffens E, Schuermans A, McNally M, Nijs S (2017) Prevention of fracture-related infection: a multidisciplinary care package. International orthopaedics 41 (12):2457-2469. doi:10.1007/s00264-017-3607-y
18. Greene LR, Mills R, Moss R, Sposato K, Vignari M (2010) The perioperative setting. Association for Professionals in Infection Control and Epidemiology. https://apic.org/wp-content/uploads/2019/02/APIC-Ortho-Guide.pdf.
19. Thakore RV, Greenberg SE, Shi H, Foxx AM, Francois EL, Prablek MA, Nwosu SK, Archer KR, Ehrenfeld JM, Obremskey WT, Sethi MK (2015) Surgical site infection in orthopedic trauma: A case-control study evaluating risk factors and cost. J Clin Orthop Trauma 6 (4):220-226. doi:10.1016/j.jcot.2015.04.004
20. Hastan D, Fokkens WJ, Bachert C, Newson RB, Bislimovska J, Bockelbrink A, Bousquet PJ, Brozek G, Bruno A, Dahlen SE, Forsberg B, Gunnbjornsdottir M, Kasper L, Kramer U, Kowalski ML, Lange B, Lundback B, Salagean E, Todo-Bom A, Tomassen P, Toskala E, van Drunen CM, Bousquet J, Zuberbier T, Jarvis D, Burney P (2011) Chronic rhinosinusitis in Europe--an underestimated disease. A GA(2)LEN study. Allergy 66 (9):1216-1223. doi:10.1111/j.1398-9995.2011.02646.x
21. Foreman A, Wormald PJ (2010) Different biofilms, different disease? A clinical outcomes study. The Laryngoscope 120 (8):1701-1706. doi:10.1002/lary.21024
22. Bendouah Z, Barbeau J, Hamad WA, Desrosiers M (2006) Biofilm formation by Staphylococcus aureus and Pseudomonas aeruginosa is associated with an unfavorable evolution after surgery for chronic sinusitis and nasal polyposis. Otolaryngology--head and neck surgery : official journal of American Academy of Otolaryngology-Head and Neck Surgery 134 (6):991-996. doi:10.1016/j.otohns.2006.03.001
23. Oomen KP, April MM (2012) Sinonasal manifestations in cystic fibrosis. International journal of otolaryngology 2012:789572. doi:10.1155/2012/789572
24. Vos R, Vanaudenaerde BM, Geudens N, Dupont LJ, Van Raemdonck DE, Verleden GM (2008) Pseudomonal airway colonisation: risk factor for bronchiolitis obliterans syndrome after lung transplantation? The European respiratory journal 31 (5):1037-1045. doi:10.1183/09031936.00128607
25. Morlacchi LC, Greer M, Tudorache I, Blasi F, Welte T, Haverich A, Mainz JG, Gottlieb J (2018) The burden of sinus disease in cystic fibrosis lung transplant recipients. Transplant infectious disease : an official journal of the Transplantation Society 20 (5):e12924. doi:10.1111/tid.12924
26. Ooi ML, Drilling AJ, Morales S, Fong S, Moraitis S, Macias-Valle L, Vreugde S, Psaltis AJ, Wormald PJ (2019) Safety and Tolerability of Bacteriophage Therapy for Chronic Rhinosinusitis Due to Staphylococcus aureus. JAMA otolaryngology-- head & neck surgery. doi:10.1001/jamaoto.2019.1191
27. Miyake MM, Bleier BS (2019) Future topical medications in chronic rhinosinusitis. International forum of allergy & rhinology 9 (S1):S32-s46. doi:10.1002/alr.22341
28. Bassetti M, Righi E, Carnelutti A (2016) Bloodstream infections in the Intensive Care Unit. Virulence 7 (3):267-279. doi:10.1080/21505594.2015.1134072
29. Pouillot F, Chomton M, Blois H, Courroux C, Noelig J, Bidet P, Bingen E, Bonacorsi S (2012) Efficacy of bacteriophage therapy in experimental sepsis and meningitis caused by a clone O25b:H4-ST131 Escherichia coli strain producing CTX-M-15. Antimicrobial agents and chemotherapy 56 (7):3568-3575. doi:10.1128/aac.06330-11
30. Capparelli R, Nocerino N, Iannaccone M, Ercolini D, Parlato M, Chiara M, Iannelli D (2010) Bacteriophage therapy of Salmonella enterica: a fresh appraisal of bacteriophage therapy. The Journal of infectious diseases 201 (1):52-61. doi:10.1086/648478
31. Gu J, Liu X, Li Y, Han W, Lei L, Yang Y, Zhao H, Gao Y, Song J, Lu R, Sun C, Feng X (2012) A method for generation phage cocktail with great therapeutic potential. PloS one 7 (3):e31698. doi:10.1371/journal.pone.0031698

32. Law N, Logan C, Yung G, Furr CL, Lehman SM, Morales S, Rosas F, Gaidamaka A, Bilinsky I, Grint P, Schooley RT, Aslam S (2019) Successful adjunctive use of bacteriophage therapy for treatment of multidrug-resistant Pseudomonas aeruginosa infection in a cystic fibrosis patient. Infection 47 (4):665-668. doi:10.1007/s15010-019-01319-0
33. LaVergne S, Hamilton T, Biswas B, Kumaraswamy M, Schooley RT, Wooten D (2018) Phage Therapy for a Multidrug-Resistant Acinetobacter baumannii Craniectomy Site Infection. Open forum infectious diseases 5 (4):ofy064. doi:10.1093/ofid/ofy064
34. Petrovic Fabijan A, Lin RCY, Ho J, Maddocks S, Ben Zakour NL, Iredell JR (2020) Safety of bacteriophage therapy in severe Staphylococcus aureus infection. Nature microbiology 5 (3):465-472. doi:10.1038/s41564-019-0634-z
35. Nir-Paz R, Gelman D, Khouri A, Sisson BM, Fackler J, Alkalay-Oren S, Khalifa L, Rimon A, Yerushalmy O, Bader R, Amit S, Coppenhagen-Glazer S, Henry M, Quinones J, Malagon F, Biswas B, Moses AE, Merril G, Schooley RT, Brownstein MJ, Weil YA, Hazan R (2019) Successful treatment of antibiotic resistant poly-microbial bone infection with bacteriophages and antibiotics combination. Clinical infectious diseases : an official publication of the Infectious Diseases Society of America. doi:10.1093/cid/ciz222
36. Schooley RT, Biswas B, Gill JJ, Hernandez-Morales A, Lancaster J, Lessor L, Barr JJ, Reed SL, Rohwer F, Benler S, Segall AM, Taplitz R, Smith DM, Kerr K, Kumaraswamy M, Nizet V, Lin L, McCauley MD, Strathdee SA, Benson CA, Pope RK, Leroux BM, Picel AC, Mateczun AJ, Cilwa KE, Regeimbal JM, Estrella LA, Wolfe DM, Henry MS, Quinones J, Salka S, Bishop-Lilly KA, Young R, Hamilton T (2017) Development and Use of Personalized Bacteriophage-Based Therapeutic Cocktails To Treat a Patient with a Disseminated Resistant Acinetobacter baumannii Infection. Antimicrobial agents and chemotherapy 61 (10). doi:10.1128/aac.00954-17
37. Vogt D, Sperling S, Tkhilaishvili T, Trampuz A, Pirnay JP, Willy C (2017) [Beyond antibiotic therapy - Future antiinfective strategies - Update 2017]. Der Unfallchirurg 120 (7):573-584. doi:10.1007/s00113-017-0374-6
38. Tkhilaishvili T, Winkler T, Muller M, Perka C, Trampuz A (2019) Bacteriophages as adjuvant to antibiotics for the treatment of periprosthetic joint infection caused by multidrug-resistant Pseudomonas aeruginosa. Antimicrobial agents and chemotherapy. doi:10.1128/aac.00924-19
39. Ferry T, Boucher F, Fevre C, Perpoint T, Chateau J, Petitjean C, Josse J, Chidiac C, L'Hostis G, Leboucher G, Laurent F (2018) Innovations for the treatment of a complex bone and joint infection due to XDR Pseudomonas aeruginosa including local application of a selected cocktail of bacteriophages. The Journal of antimicrobial chemotherapy 73 (10):2901-2903. doi:10.1093/jac/dky263
40. Ferry T, Leboucher G, Fevre C, Herry Y, Conrad A, Josse J, Batailler C, Chidiac C, Medina M, Lustig S, Laurent F (2018) Salvage Debridement, Antibiotics and Implant Retention ("DAIR") With Local Injection of a Selected Cocktail of Bacteriophages: Is It an Option for an Elderly Patient With Relapsing Staphylococcus aureus Prosthetic-Joint Infection? Open forum infectious diseases 5 (11):ofy269. doi:10.1093/ofid/ofy269
41. Furfaro LL, Payne MS, Chang BJ (2018) Bacteriophage Therapy: Clinical Trials and Regulatory Hurdles. Frontiers in cellular and infection microbiology 8:376. doi:10.3389/fcimb.2018.00376
42. Dabrowska K, Abedon ST (2019) Pharmacologically Aware Phage Therapy: Pharmacodynamic and Pharmacokinetic Obstacles to Phage Antibacterial Action in Animal and Human Bodies. Microbiology and molecular biology reviews : MMBR 83 (4). doi:10.1128/mmbr.00012-19
