# Supplementary material for: Bacteriophage Therapy for Difficult-to-Treat Infections: The Implementation of a Multidisciplinary Phage Task Force (The PHAGEFORCE Study Protocol)
Source: Viruses. 2021 Aug 5;13(8):1543. doi: 10.3390/v13081543 (PMC8402896; doi:10.3390/v13081543)
Supplement: Supplementary file 1 [file viruses-13-01543-s001.zip › table1_v2.pdf]

| | Prior to phage therapy | | During phage therapy | | | | | | | | | | After cessation of phage therapy | | | | | | | | | |
|---|---|---|---|---|---|---|---|---|---|---|---|---|---|---|---|---|---|---|---|---|---|---|
| | Inclusion[1] | D0[2] | D1 | D2 | D3 | D4 | D5 | D6 | D7 | D8 | D9 | D10 | W1 | W2 | W3 | W4 | W6 | W8 | W10 | W12/3 months | 6 months | 12 months |
| **Musculoskeletal infection** | | | | | | | | | | | | | | | | | | | | | | |
| ICF[1] | X | | | | | | | | | | | | | | | | | | | | | |
| Demographics, MH, Conmeds | X | | | | | | | | | | | | | | | | | | | | | |
| ASA score | X | | | | | | | | | | | | | | | | | | | | | |
| Physical exam[3] | | X | X | X | X | X | X | X | X | X | X | X | | X | | X | X | X | X | X | upon indication | |
| Blood analysis | | X | X | X | | X | | | X | | | X | | X | | X | X | X | X | X | upon indication | |
| Serum collection[4] | | X | X | X | | X | | | X | | | X | | X | | X | X | X | X | X | | |
| Parameter collection[3] | | X | X | X | X | X | X | X | X | X | X | X | | X | | X | X | X | X | X | upon indication | |
| Deep tissue cultures[4] | X[5] | | X[6] | upon indication | | | | | | | | | upon indication | | | | | | | | | |
| Draining fluid[4] | | | X | X | X | X | X | X | X | X | X | X | | | | | | | | | | |
| PROMIS global health | | X | | | | | | | | | | X | | | | | | | | X | X | X |
| PROMIS pain interference | | X | | | | | | | | | | X | | | | | | | | X | X | X |
| PROMIS physical function | | X | | | | | | | | | | X | | | | | | | | X | X | X |
| Radiological evaluation | | X | | | | | | | | | | X | | | | | | | | upon indication | | |
| **Chronic rhinosinusitis** | | | | | | | | | | | | | | | | | | | | | | |
| ICF[1] | X | | | | | | | | | | | | | | | | | | | | | |
| Demographics, MH, Conmeds | X | | | | | | | | | | | | | | | | | | | | | |
| Physical exam including nasal endoscopy[3] | X | X | X | | | | X | | | | | | X | | | X | | | | X | | |
| Blood analysis | X | | X | | | | X | | | | | | X | | | X | | | | X | | |
| Serum collection[4] | X | | X | | | | X | | | | | | X | | | X | | | | X | | |
| Parameter collection[3] | | X | X | | | | X | | | | | | X | | | X | | | | X | | |
| Nasal swabs[4] | X[5] | X | | | | | X | | | | | | X | | | X | | | | X | | |
| PROMIS global health | X | | | | | | | | | | | | X | | | X | | | | X | | |
| PROMIS pain interference | X | | | | | | | | | | | | X | | | X | | | | X | | |
| SNOT-22 questionnaire | X | | | | | | | | | | | | X | | | X | | | | X | | |
| VAS score | X | | | | | | | | | | | | X | | | X | | | | X | | |
| CT scan (Lund-Mackay score) | | ≤3 months before start of phage therapy | upon indication | | | | | | | | | | upon indication | | | | | | | | | |
| Nasal endoscopy (Lund-Kennedy, Modified Davos scoring system) | ≤3 months prior to inclusion | | X | | | | X | | | | | | X | | | X | | | | X | | |
| Allergy testing | X or recent testing <5 years prior to inclusion | | upon indication | | | | | | | | | | upon indication | | | | | | | | | |
| Smell testing (Sniffin' sticks) | X | | | | | | | | | | | | | | | | | | | X | | |
| **Sepsis** | | | | | | | | | | | | | | | | | | | | | | |
| ICF[1] | X | | | | | | | | | | | | | | | | | | | | | |
| Demographics, MH, Conmeds | X | | | | | | | | | | | | | | | | | | | | | |
| Physical exam[3] | | X | X | X | X | X | X | X | X | X | X | X | X | X | X | X | X | X | X | X | upon indication | |
| Blood analysis | X | X | X | X | X | X | X | X | X | X | X | X | X | X | X | X | X | X | X | X | upon indication | |
| Serum collection[4] | X | X | X | X | X | X | X | X | X | X | X | X | X | X | X | X | X | X | X | X | | |
| Parameter collection[3] | X | X | X | X | X | X | X | X | X | X | X | X | X | X | X | X | X | X | X | X | upon indication | |
| Hemocultures[4] | X[5] | X | X | X | X | X | X | X | X | X | X | X | X | X | X | X | X | X | X | X | upon indication | |
| SOFA score | X | X | X | X | X | X | X | X | X | X | X | X | daily until discharge | | | | | | | | | |

**Supplementary table 1.** Flowchart of tests and sampling performed in patients receiving phage therapy. D: day; W: week; ICF: Informed Consent Form; MH: medical history; Conmeds: concomitant medications; ASA: American Society of Anesthesiologists; PROMIS: Patient-Reported Outcome Measurement Information System; SNOT: Sino-Nasal Outcome Test; VAS: Visual Analogue Scale, SOFA: Sequential Organ Failure Assessment.

[1] After positive evaluation by the CBL, informed consent is asked from the patient, either at the outpatient clinic or at the hospital ward.

[2] Day 0 is defined as the day prior to when phage therapy is started or the same day, prior to the first administration.

[3] A physical exam and parameter collection will be performed before and after each phage administration for MSI and sepsis patients. For CRS patients, these tests will be performed at inclusion, before and after the first administration (at the outpatient clinic), and at each outpatient visit .

[4] Depending on the sample type, all pathogens isolated during the duration of the study will be stored at -80°C for further analysis (deep tissue cultures, nasal swabs, sputum samples, hemocultures), and/or, all phages isolated during the duration of the study will be sequenced (serum samples, draining fluid, nasal swabs).

[5] If no cultures or isolated pathogens are available at the time of inclusion, cultures (deep tissue cultures for MSI patients, nasal swabs for CRS patients and hemocultures for sepsis patients) will be taken at inclusion. The isolated pathogens are sent for susceptibility testing against the available phages. Only if the causative pathogens are susceptible to the available phages, phage therapy can be started.

[6] Phage therapy for MSI patients is applied via a draining system that is placed during surgery. During this procedure, deep tissue cultures are taken again.
